# Dynamic evolution of selenocysteine utilization in bacteria: a balance between selenoprotein loss and evolution of selenocysteine from redox active cysteine residues

**DOI:** 10.1186/gb-2006-7-10-r94

**Published:** 2006-10-20

**Authors:** Yan Zhang, Hector Romero, Gustavo Salinas, Vadim N Gladyshev

**Affiliations:** 1 Department of Biochemistry, University of Nebraska, 1901 Vine street, Lincoln, NE 68588-0664, USA; 2 Laboratorio de Organización y Evolución del Genoma, Laboratorio de Organización y Evolución del Genoma, Dpto de Biología Celular y Molecular, Instituto de Biología, Facultad de Ciencias, Iguá 4225, Montevideo, CP 11400, Uruguay; 3 Cátedra de Inmunología, Facultad de Química/Ciencias, Instituto de Higiene, Avda A Navarro 3051, Montevideo, CP 11600, Uruguay

## Abstract

**Background:**

Selenocysteine (Sec) is co-translationally inserted into protein in response to UGA codons. It occurs in oxidoreductase active sites and often is catalytically superior to cysteine (Cys). However, Sec is used very selectively in proteins and organisms. The wide distribution of Sec and its restricted use have not been explained.

**Results:**

We conducted comparative genomics and phylogenetic analyses to examine dynamics of Sec decoding in bacteria at both selenium utilization trait and selenoproteome levels. These searches revealed that 21.5% of sequenced bacteria utilize Sec, their selenoproteomes have 1 to 31 selenoproteins, and selenoprotein-rich organisms are mostly *Deltaproteobacteria *or *Firmicutes*/*Clostridia*. Evolutionary histories of selenoproteins suggest that Cys-to-Sec replacement is a general trend for most selenoproteins. In contrast, only a small number of Sec-to-Cys replacements were detected, and these were mostly restricted to formate dehydrogenase and selenophosphate synthetase families. In addition, specific selenoprotein gene losses were observed in many sister genomes. Thus, the Sec/Cys replacements were mostly unidirectional, and increased utilization of Sec by existing protein families was counterbalanced by loss of selenoprotein genes or entire selenoproteomes. Lateral transfers of the Sec trait were an additional factor, and we describe the first example of selenoprotein gene transfer between archaea and bacteria. Finally, oxygen requirement and optimal growth temperature were identified as environmental factors that correlate with changes in Sec utilization.

**Conclusion:**

Our data reveal a dynamic balance between selenoprotein origin and loss, and may account for the discrepancy between catalytic advantages provided by Sec and the observed low number of selenoprotein families and Sec-utilizing organisms.

## Abstract 2

Comparative genomics and evolutionary analyses to examine the dynamics of selenocysteine utilization in bacteria reveal a dynamic balance between selenoprotein origin and loss.

## Background

Selenium, an essential trace element for many organisms in the three domains of life, is present in proteins in the form of selenocysteine (Sec) residue [1-4]. Sec, known as the 21st naturally occurring amino acid, is co-translationally inserted into proteins by recoding opal (UGA) codons. These UGA codons are recognized by a complex molecular machinery, known as selenosome, which is superimposed on the translation machinery of the cell. Although the Sec insertion machinery differs in the three domains of life, its origin appears to precede the domain split [1,2,5-8].

The mechanism of Sec insertion in response to UGA in bacteria has been most thoroughly elucidated in *Escherichia coli *[1,2,9-11]. Briefly, selenoprotein mRNA carries a selenocysteine insertion sequence (SECIS) element, immediately downstream of Sec-encoding UGA codon [2,3,12]. The SECIS element binds the Sec-specific elongation factor (SelB, the *selB *gene product) and forms a complex with tRNA^Sec ^(the *selC *gene product), whose anticodon matches the UGA codon. tRNA^Sec ^is initially acylated with serine by a canonical seryl-tRNA synthetase and is then converted to Sec-tRNA^Sec ^by Sec synthase (SelA, the *selA *gene product). SelA utilizes selenophosphate as the selenium donor, which in turn is synthesized by selenophosphate synthetase (SelD, the *selD *gene product).

In addition, in some organisms selenophosphate is also a selenium donor for biosynthesis of a modified tRNA nucleoside, namely 5-methylaminomethyl-2-selenouridine (mnm^5^Se^2^U), which is present at the wobble position of tRNA^Lys^, tRNA^Glu^, and tRNA^Gln ^anticodons [13]. The proposed function of mnm^5^Se^2^U in these tRNAs involves codon-anticodon interactions that help base pair discrimination at the wobble position and/or translation efficiency [14]. A 2-selenouridine synthase (YbbB, the *ybbB *gene product) is necessary to replace a sulfur atom in 2-thiouridine in these tRNAs with selenium [15]. In addition, selenium is utilized in the form of co-factor in certain molybdenum-containing enzymes [16,17].

The Sec-decoding trait is the main biologic system of selenium utilization, as evidenced by its distribution in living organisms. Sec is present in the active sites of functionally diverse selenoproteins, most of which exhibit redox function. It has been reported that Sec can greatly increase the catalytic efficiency of selenoenzymes as compared with their cysteine (Cys)-containing homologs [18]. Despite this selective advantage and its dedicated biosynthesis and decoding machinery, Sec is a rare amino acid. The selenoproteome of a given Sec-incorporating organism is represented by a small number of protein families. Twenty-six eukaryotic and 27 prokaryotic selenoprotein families (including 25 bacterial selenoprotein families) have previously been reported [19-21], and additional selenoproteins could probably be identified by computational analyses of large sequence datasets [22].

Recent phylogenetic analyses of components of both Sec-decoding and selenouridine traits in completely sequenced bacterial genomes have provided evidence for a highly mosaic pattern of species that incorporate Sec, which can be explained as the result of speciation, differential gene loss and horizontal gene transfer (HGT), indicating that neither the loss nor the acquisition of the trait is irreversible [13]. However, it is still unclear why this amino acid is only utilized by a subset of organisms. Even more puzzling is the fact that many organisms that are able to decode Sec use this amino acid only in a small set of proteins or even in a single protein. It would be interesting to determine whether there are environmental factors that specifically affect selenoprotein evolution.

The aim of this work was to address these questions by analyzing evolution of selenium utilization traits (Sec decoding and selenouridine utilization) and selenoproteomes in bacteria. We have performed phylogenetic analyses of key components of these traits (SelA, SelB, SelD, and YbbB) and analyzed 25 selenoprotein families in bacterial genomes for which complete or nearly complete sequence information is available. The data suggest that in most selenoprotein families, especially those containing rare selenoproteins and widespread Cys-containing homologs, selenoproteins have evolved from a Cys-containing ancestor. In addition, the majority of selenoprotein-rich organisms are anaerobic hyperthermophiles that belong to a small number of phyla. Selenoprotein losses could be detected in a number of sister genomes of selenoprotein-rich organisms. These observations revealed a dynamic and delicate balance between Sec acquisition and selenoprotein loss, and may partially explain the discrepancy between catalytic advantages offered by Sec and its limited use in nature. This balance is seen at three levels: loss and acquisition of the Sec-decoding trait itself, with the former as a predominant route; emergence/loss of selenoprotein families; and Cys-to-Sec or Sec-to-Cys replacements in different selenoprotein families.

## Results

### Distribution of selenium utilization traits in bacteria

**Figure 1:**
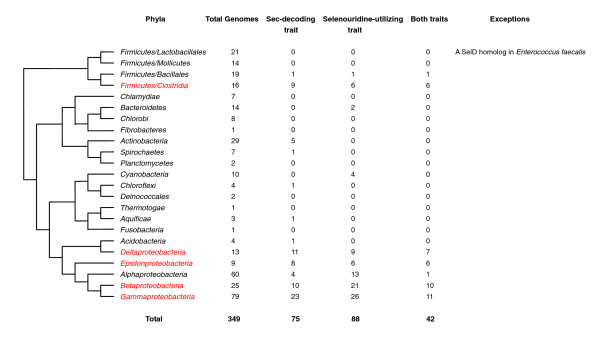
Distribution of selenium utilization traits in different bacterial taxa. The tree is based on a highly resolved phylogenetic tree of life derived from a concatenation of 31 orthologs occurring in 191 species with sequenced genomes [23]. We simplified the complete tree and only show the bacterial branches. Phyla containing the majority of Sec-decoding organisms are shown in red.

Sequence analysis of bacterial genomes revealed wide distribution of genes encoding key components of Sec-decoding (SelA/SelB/SelC/SelD) and selenouridine-utilizing (SelD/YbbB) machinery. We identified 75 Sec-decoding (21.5% of all sequenced genomes) and 88 selenouridine-utilizing (25.2% of all sequenced genomes) organisms. Figure 1 shows the distribution of the two selenium utilization traits in different bacterial taxa based on a highly resolved phylogenetic tree of life [23]. It has been proposed that SelB is the signature of the Sec-decoding and YbbB of the selenouridine traits [13]. SelD is required for both pathways and this protein defines the overall selenium utilization trait. Figure 1 shows that, except for the phyla containing only one or two sequenced genomes (for example, *Deinococcales, Fibrobacteres*, and *Planctomycetes*), SelD is present in nearly all bacterial phyla with the exception of *Chlamydiae, Chlorobi*, and *Firmicutes/Mollicutes*. This observation suggests that selenium may be used by most bacterial lineages and that selenium utilization is an ancient trait that once was common to all or almost all species in this domain of life. Among SelD-containing species, the majority of Sec-decoding organisms (having SelA and SelB) belong to *Proteobacteria *and *Firmicutes*, especially *Betaproteobacteria, Deltaproteobacteria, Epsilonproteobacteria, Gammaproteobacteria *and *Firmicutes/Clostridia *subdivisions, in which the Sec-decoding trait was found in at least 10 genomes or 50% of all sequenced genomes. In contrast, the Sec-decoding trait was not detected among *Bacteroidetes *and *Cyanobacteria*. It is possible that selenoprotein-containing organisms in these phyla have not yet been sequenced, or that the trait was lost at the base of these phyla. The selenouridine-utilizing trait was found to be absent in all sequenced organisms of *Actinobacteria, Spirochaetes, Chloroflexi, Aquificae *and *Acidobacteria*, some of which have selenoproteins, and present in *Bacteroidetes *and *Cyanobacteria*, some of which lack selenoproteins; this indicates a relatively independent relationship between the two selenium utilization traits. Nevertheless, significant overlap between the presence of Sec and selenouridine traits observed in the present study suggests that one selenium utilization trait may facilitate acquisition/maintenance of the second because of the common gene involved (SelD).

A unique exception was the detection of an orphan SelD without any other known components of selenium utilization traits or genes encoding selenoproteins in the complete genome of *Enterococcus faecalis*, which is the only SelD-containing member of the *Firmicutes/Lactobacillales *subdivision. A similar situation was also observed in the archaeal plasmid, *Haloarcula marismortui *plasmid pNG700. The presence of *selD *in organisms that lacked known selenium utilization traits suggested that there might be a third trait dependent on SelD. In addition to Sec-containing proteins and selenouridine-containing tRNAs, selenium occurs in several bacterial molybdenum-containing oxidoreductases in the form of an undefined co-factor [17,24-26]. However, no genes have been linked either to biosynthesis of this selenium species or to insertion of the selenium co-factor into proteins.

Several SelA homologs were also found in organisms that lacked the Sec-decoding trait. In addition, a recent structural and functional investigation into an archaeal SelA homolog revealed that it lacks SelA activity [27]. These findings indicate that SelA might have acquired a new function in these organisms.

### Phylogenetic analysis of selenium utilization traits

Seventy-five SelA (excluding nine homologs in organisms lacking selenoproteins), 75 SelB, 127 SelD, and 88 YbbB sequences from different bacterial species were used to build protein-specific phylogenetic trees. Most branches were consistent with the evolutionary relationships between bacterial species. However, some HGT events could also be observed in these trees  ( Additional data file 2 [Figure S1]).

**Figure 2:**
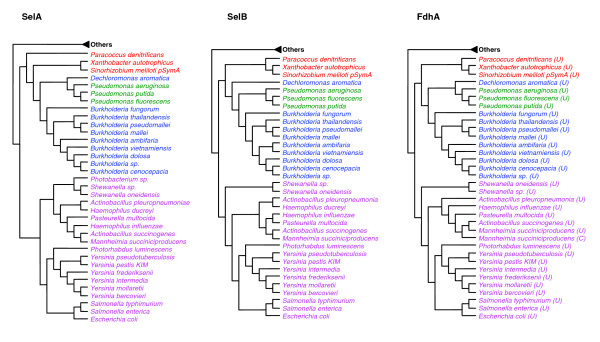
Phylograms of SelA, SelB, and FdhA sequences from *Alphaproteobacteria, Betaproteobacteria*, and *Gammaproteobacteria*. Organisms and phyla are shown by different colors. Red indicates *Alphaproteobacteria*, blue indicates *Betaproteobacteria*, green indicates *Gammaproteobacteria/Pseudomonadales*, and pink indicates other *Gammaproteobacteria*. In the FdhA phylogram, U represents Sec-containing sequences and C Cys-containing sequences.

In addition to the previously reported HGT of the entire Sec-decoding trait and selenoproteins observed in *Photobacterium profundum *(*Gammaproteobacteria*) and *Treponema denticola *(*Spirochaetes*) [13], the topologies of SelA and SelB phylogenetic trees reveal that the *Pseudomonadale *sequences are within the *Alphaproteobacteria-Betaproteobacteria *node, and not - as expected for vertical descent - within the *Gammaproteobacteria *node(Figure 2). This suggests that there is another HGT event. In addition, the topology of formate dehydrogenase α subunit (FdhA) tree, which is the only selenoprotein in *Pseudomonadales*, is consistent with an HGT event (Figure 2). We further analyzed the genomic organization of the Sec-decoding trait and *fdhA *genes in these genomes. The *selA, selB*, and *selC *genes were organized in operons and the *fdhA *gene was very close to or even flanked the *selA-selB-selC *operon. Our data strongly suggest that both the Sec-decoding trait (*selA, selB*, and *selC*) and *fdhA *of *Pseudomonadales *were acquired by HGT. Evolution of *selD *might be independent from other components involved in Sec decoding; selenophosphate is required for two different selenium utilization traits that exhibit overlapping but distinct phylogenetic distribution. Indeed, phylogenetic analyses indicate that *Pseudomonadales *acquired the selenouridine trait by vertical descent; furthermore, as in many other species containing both traits, *selD *and *ybbB *are arranged in an operon. These observations suggest that in the presence of *selD *(utilized by selenouridine), Sec-decoding could have been acquired by HGT of *selA, selB *and *selC*, as well as the first selenoprotein gene. This step-wise evolution to selenium utilization is a parsimonious and plausible route for acquisition of an additional selenium-dependent trait from an already existing one, and could have helped to spread both traits vertically or laterally during evolution. The selenouridine biosynthesis trait was also analyzed as described for the Sec trait. Frequent HGT events were observed, but co-transfer of both traits was not detected.

### Distribution and phylogenetic analysis of selenoprotein families

We analyzed 25 known bacterial selenoprotein families (including SelD), which were represented by 285 selenoprotein sequences in sequenced bacterial genomes. Among them, 18 families were orthologs of thiol-based redox proteins. Distribution of sequences for each selenoprotein family is shown in Table 1. FdhA and SelD are the most widespread selenoproteins, and at least one of these proteins was present in each selenoprotein-containing organism. FdhA was found in 67 out of 75 (89.3%) organisms that utilize Sec.

Analysis of distribution of selenoprotein families in different bacterial phyla showed the high diversity of bacterial selenoproteomes. Most bacterial phyla/branches contained only one to three selenoprotein families (Table 2). However, three separate selenoprotein family-rich phyla were identified: *Deltaproteobacteria *(22 families), *Firmicutes*/*Clostridia *(16 families), and *Actinobacteria *(12 families). A total of 198 selenoproteins belonging to all 25 families were identified in these three phyla, which accounted for 69.5% of all detected selenoprotein sequences, suggesting high Sec usage in the three phyla. Moreover, 18 selenoprotein-rich organisms (number of selenoproteins six or greater) were identified in most *Deltaproteobacteria *(10/11) and *Firmicutes/Clostridia *(6/9), as well as one *Actinobacterium *(*Symbiobacterium thermophilum*) and one *Spirochaete *(*Treponema denticola*; Table 3).

**Figure 3:**
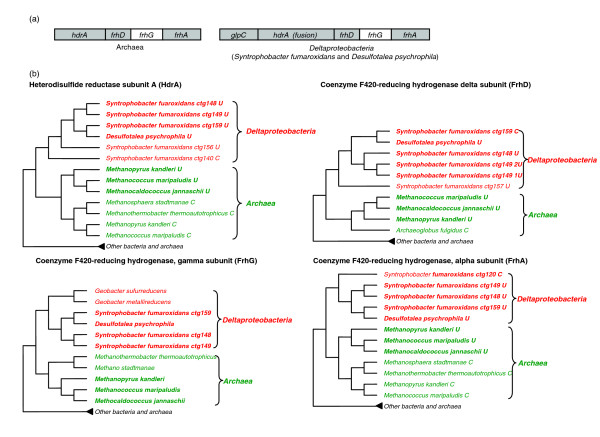
Organization and phylogenetic analysis of components of the archaeal four-gene and bacterial five-gene operons. **(a) **Organization of operons in archaea and bacteria. Selenoprotein genes are shaded. **(b) **Phylograms of different proteins in these operons. Red indicates *Deltaproteobacteria*, and green indicates Archaea. Organisms containing the four-gene or five-gene operon are shown in bold. The branch separating other archaea and bacteria in the trees has been shortened for illustration purposes. C, Cys-containing; FrhA, coenzyme F420-reducing hydrogenase α subunit; FrhD, coenzyme F420-reducing hydrogenase δ subunit; FrhG, coenzyme F420-reducing hydrogenase γ subunit; GlpC, Fe-S oxidoreductase; HdrA, heterodisulfide reductase subunit A; U, Sec-containing.

One deltaproteobacterium, namely *Syntrophobacter fumaroxidans*, was identified that contained 31 selenoprotein genes, the largest selenoproteome reported to date, including those of eukaryotes. Multiple copies of heterodisulfide reductase subunit A (HdrA), coenzyme F420-reducing hydrogenase δ subunit (FrhD), and coenzyme F420-reducing hydrogenase α subunit (FrhA) were found in this organism. These three selenoprotein families are present in all three known selenoprotein-containing archaea (*Methanocaldococcus jannaschii, Methanococcus maripaludis*, and *Methanopyrus kandleri*) and in several bacteria [19,28]. We analyzed the genomic locations of these three selenoprotein families in both archaeal and bacterial genomes. In archaea, genes of Sec-containing HdrA, FrhD, and FrhA are always present with coenzyme F420-reducing hydrogenase γ subunit (FrhG, not a selenoprotein), in an operon *hdrA-frhD-frhG-frhA*. Surprisingly, these four genes were also found to be clustered in some *Deltaproteobacteria*, especially *Syntrophobacter fumaroxidans*, which contained three similar five-gene operons. These operons also had an additional selenoprotein family, namely Fe-S oxidoreductase (GlpC), which is absent in Sec-decoding archaea (Figure 3a). Although additional Sec- and Cys-containing homologs were also present, phylogenetic analysis of HdrA, FrhD, FrhG, and FrhA sequences in these operons showed that sequences from all Sec-decoding archaea and *Syntrophobacter fumaroxidans *clustered in one sub-branch in each evolutionary tree (Figure 3b). Another member of *Deltaproteobacteria*, namely *Desulfotalea psychrophila*, which contains the same five-gene operon as that in *Syntrophobacter fumaroxidans*, was also represented in these sub-branches. The remaining archaeal and bacterial sequences corresponded to more distant subfamilies. This topology is consistent with the idea that the whole *hdrA-frhD-frhG-frhA *operon was transferred between archaea and *Deltaproteobacteria*. Moreover, *Syntrophobacter fumaroxidans *is an obligate anaerobe, which degraded propionate in syntrophic association with methanogens [29]. In contrast to archaea, all *hdrA *genes in the bacterial operon were clustered with themselves with or without insertion of an additional gene of unknown function in between (*hdrA-hdrA *gene and *hdrA_N-unknown-hdrA_C *gene, respectively). These data revealed a complex and highly dynamic evolutionary process of selenoproteins in *Deltaproteobacteria*.

### Origin and loss of selenoproteins via Sec/Cys conversions

Distribution of Sec-/Cys-containing sequences in organisms containing and lacking the Sec-decoding trait is shown in Additional data files 1 (Table S1) and 2 (Figure S2). In most selenoprotein families, the number of Sec-containing sequences was much smaller than that of Cys-containing homologs. The occurrence of Sec- and Cys-containing homologs suggested a close evolutionary relationship between these proteins. However, it is not known whether Sec evolves from Cys residues or Cys from Sec. In addition, if both conversion types are possible, then it which is the predominant one is also unknown.

**Figure 4:**
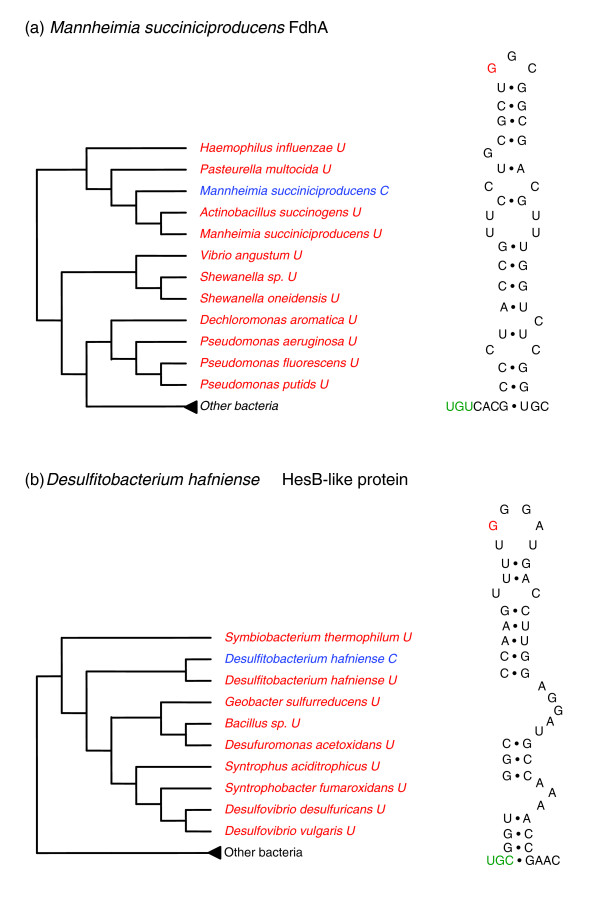
Phylograms and putative remnant bacterial SECIS-like structures in two Cys-containing sequences evolved from Sec-containing homologs. In the phylograms, organisms containing the Sec-containing sequences are shown in red, and organisms containing the Cys-containing homologs are shown in blue. In the bacterial SECIS-like structures, codons for Cys are shown in green and the conserved G in the apical loop is shown in red. **(a) ***Mannheimia succiniciproducens *FdhA. **(b) ***Desulfitobacterium hafniense *HesB-like protein. C, Cys-containing; SECIS, selenocysteine insertion sequence; U, Sec-containing.

To address these questions, we analyzed evolutionary relationships between Sec-containing and Cys-containing forms in each selenoprotein family, except glycine reductase selenoprotein A (GrdA), which had no known Cys-containing homologs. Not all selenoproteins were informative in this analysis, because in the majority of phylogenetic trees the evolutionary origin of sequences could not be reliably assessed. However, this analysis revealed 33 events in 17 selenoprotein families that corresponded to Cys-to-Sec conversions (Cys→Sec). Most of these events were detected in various selenoprotein families containing few selenoprotein sequences. Interestingly, 15 of these 17 selenoprotein families had a common feature; they were homologs of thiol-based redox proteins, which contained UxxC, CxxU or TxxU redox motifs. In contrast, only 15 events were detected that corresponded to Sec-to-Cys conversions (Sec→Cys). Moreover, these events occurred only in four families (see the two middle columns in Table 1). Among Cys-containing homologs that probably evolved from selenoproteins, 11 occurred in selenoprotein-containing organisms (these organisms lost a particular selenoprotein but not the ability to decode Sec) and some contained remnant bacterial SECIS-like structures downstream of the Cys codons, providing further evidence in support of their selenoprotein ancestors (see examples in Figure 4).

The majority of the detected Sec→Cys conversions (66.7%) were associated with the FdhA and SelD families (46.7% for FdhA and 20% for SelD). In contrast, no Cys→Sec events were observed in these two families, which are by far the two most abundant selenoprotein families in the bacterial domain. An attractive hypothesis is that the Sec-decoding trait largely co-evolved with the Sec-containing FdhA. In most families containing rare selenoproteins and widespread Cys-containing homologs, the selenoproteins evolved from Cys-containing ancestors; however, these events could only occur in organisms that already possessed the Sec-decoding trait and FdhA. In the absence of FdhA, SelD could be involved in maintaining the Sec-decoding trait (perhaps to sustain efficient selenouridine formation), as suggested by the facts that all Sec-decoding organisms that lack FdhA have Sec-containing SelD and that most of them possess the selenouridine trait.

### Identification of selenoprotein loss events in sister species

Sec is normally a much more reactive residue than Cys [30-32]. Because it provides catalytic advantage over Cys in certain redox enzymes, Sec may be expected to have a widespread occurrence. In addition, the higher rate of Cys→Sec conversions compared with that of Sec→Cys events would result in increased utilization of Sec during evolution. However, the number of selenoprotein families identified to date is small, and no clear explanation is available for this discrepancy.

We analyzed the evolutionary trends in different selenoprotein families by assessing the occurrence of orthologous selenoproteins in sister and relatively distant organisms selected from the same phylum (see Materials and methods, below). If only one of two (or more) sister genomes and at least two distant genomes carried orthologous Sec/Cys-containing sequences, then a selenoprotein gene loss event in the sister genomes could be inferred. The last column in Table 1 shows putative evolutionary scenarios for each selenoprotein family. Although many selenoproteins were not informative in identifying the events associated with selenoprotein loss (there were 201 widespread selenoproteins and 46 selenoproteins in which selenoprotein loss and origin events could not be distinguished), we could identify 38 events of selenoprotein loss in 12 selenoprotein families (Table 4). Among them, 26 occurred in different subgroups of *Firmicutes/Clostridia*, eight in *Deltaproteobacteria*, and four in *Actinobacteria*, which are the three selenoprotein-rich phyla (Additional data file 1 [Table S3]). No events of selenoprotein loss were observed in other phyla.

## Discussion

Although much effort has previously been devoted to identifying selenoprotein genes and Sec insertion machinery, evolution of selenium utilization traits remained unclear. Some primary considerations concerning the phylogeny of Sec incorporation and the evolution of Sec have previously been proposed [33]. The major usage of selenium in nature appears to be in co-translational incorporation of Sec into selenoproteins. In addition, 2-selenouridine, a modified tRNA nucleotide in the wobble position of anticodons of some tRNAs, has been identified as a second selenium utilization trait [13]. A common feature between the two selenium utilization traits is that both use selenophosphate as the selenium donor. Therefore, SelD is considered to be a general signature for selenium utilization.

In the present study we scrutinized, using various methods, homologous Sec- and Cys-containing sequences evolved in bacterial genomes, which provided important new insights into the dynamic evolution of selenium utilization in bacteria. The widespread taxa distribution of selenium utilization traits agreed with the idea that selenium could be used by various species in almost all bacterial phyla. However, among all sequenced bacterial genomes, only 21.5% possess the Sec-decoding trait and 25.2% the selenouridine-utilizing trait, suggesting that most organisms lost the ability to utilize Sec or selenouridine. It should be noted that many Sec-decoding organisms also possessed the selenouridine-utilizing trait and *vice versa*, suggesting that the two traits might have evolved under similar environmental conditions (for example, selenium supply) or could influence evolution of each other. However, the occurrence of many organisms containing only one of these traits indicates that selenium availability is not the sole factor responsible for acquisition or loss of either trait, and suggests a relatively independent and complementary relationship between the two selenium utilization traits. The presence of SelD as a single selenoprotein in several YbbB-containing species reinforces the idea that the traits might have a complementary relationship (specifically, the Sec-decoding trait might be maintained for SelD, which in turn supports both itself and selenouridine synthesis). In addition, the presence of an 'orphan *selD*' (one that is not associated with either trait) in both bacteria and archaea raised the possibility of a third, currently unknown selenium utilization trait.

We built the phylogenetic trees for both the components of selenium utilization traits and selenoproteins by several independent methods. The topologies of these inferred trees were supported by most individual trees. In addition, phylogenies of SECIS elements in different bacterial selenoprotein genes were also consistent with those of selenoproteins (data not shown), suggesting that both SECIS elements and selenoproteins have similar evolutionary trends.

**Figure 5:**
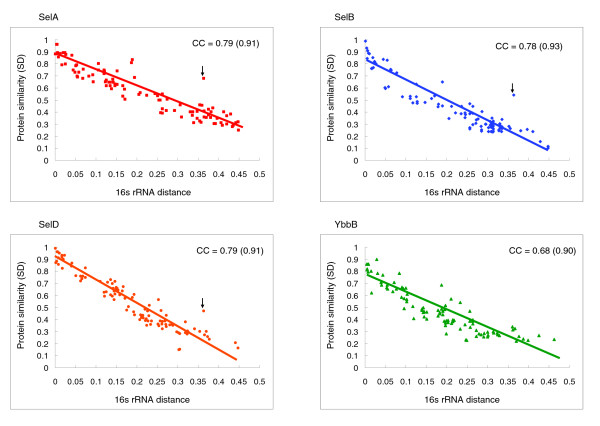
Evolutionary divergence of components of two selenium utilization traits extracted from the datasets identified in this work. Each graph contains 100 randomly selected organism pairs (points). The protein similarity (sequence divergence [SD]) of each component usually changes proportionally with the phylogenetic distance. Correlation coefficient (CC) is shown (number in the parentheses shows an updated CC after removing horizontal gene transfer [HGT] events). Points that are located above and distant from the reference line suggest potential HGT events. A reported HGT organism pair (*Photobacterium profundum *and *Treponema denticola*) of the Sec-decoding trait is shown with arrows for reference.

To establish the correspondence between the inferred phylogenies for the components of the two selenium utilization traits and the general evolutionary trend, we measured, for each pair of organisms, the correlation between the similarity of orthologous pairs and that of the 16S rRNAs (as controls). The correlation coefficient was 0.68-0.79 (Figure 5). After removing the HGT cases, all correlation coefficients were even higher (≥ 0.9). The data suggest that the inferred phylogenetic trees are consistent with the evolutionary distance derived from 16S rRNAs, and that selenium utilization systems in most bacterial species were inherited from a common ancestor in the same phylogenetic lineage.

HGT events have contributed to the evolution of Sec-decoding or selenouridine-utilizing traits. However, detection of HGT of the entire trait is difficult, especially for the Sec-decoding trait, because these events are rare. In our study, besides the HGT event previously reported for the Sec-decoding trait [13], we found that all Sec-decoding organisms in *Alphaproteobacteria, Betaproteobacteria*, and *Gammaproteobacteria/Pseudomonadales *possess similar *selA-selB-selC *operons and a neighboring *fdhA *gene, which encodes the only selenoprotein in these organisms (Figure 2). Our data provide support for the idea that a Sec-decoding HGT event can occur only if *selA, selB*, and *selC *genes are organized in a cluster and the transfer event is accompanied by co-transfer of at least one selenoprotein gene (most often *fdhA*, or *selD *if *fdhA *is absent). In addition, because SelD and YbbB are the only known components of the selenouridine-utilizing trait and their genes almost always form an operon, additional co-transfer events could be observed (although we did not detect examples of the HGT of both traits). In some phyla both selenoprotein-containing organisms and sister organisms lacking selenoproteins possess *selD *and *ybbB*; this fact suggests that evolution of SelD is relatively independent from other components of the Sec-decoding trait.

That either FdhA or SelD were present in every selenoproteome supports the idea that one or both of these two selenoprotein families are largely responsible for maintaining the Sec-decoding trait. *Deltaproteobacteria, Firmicutes/Clostridia*, and *Actinobacteria *were three selenoprotein family rich phyla, which had all 25 selenoprotein families and represented 17 out of 18 (94.4%) selenoprotein-rich organisms. The families containing rare selenoproteins (with number of selenoproteins below five) were only present in *Deltaproteobacteria *and *Firmicutes/Clostridia*, suggesting an active evolution of new selenoproteins in these two separate phyla. Considering the bias of distribution of sequenced bacterial genomes, additional selenoprotein-rich phyla or organisms might be identified in future.

A total of 31 known selenoproteins were found in *Syntrophobacter fumaroxidans *(*Deltaproteobacteria*), which is the largest selenoproteome reported thus far. This organism has multiple *glpC*-*hdrA-frhD-frhG-frhA *operons. Phylogenetic analyses of the genes in these operons suggested that the *hdrA-frhD-frhG-frhA *cluster was laterally transferred between Sec-decoding *Archaea *and *Deltaproteobacteria *(Figure 3). Compared with other lateral gene transfers between archaea and bacteria [34], selenoprotein gene transfers would be more difficult because of different mechanisms of Sec insertion into polypeptide chains [9,18,29]. No remnant bacterial-type SECIS structures could be found in archaeal selenoprotein genes or archaeal-type SECISes in bacterial selenoprotein genes. However, *Deltaproteobacteria *contained a five-gene operon which included GlpC, another selenoprotein family, in addition to the genes present in Sec-decoding archaea; also, complex evolutionary processes including gene duplications and gene fusion events involving *hdrA *were observed in *Deltaproteobacteria*. These facts suggest that *Deltaproteobacteria *might have gained the original four-gene operon from Sec-decoding archaea. Coherent clustering of selenoprotein genes in Sec-decoding archaea and *Deltaproteobacteria*, and the absence of the same operon in closely related organisms indicate that this lateral transfer might have happened only recently.

The analysis of selenoproteins and the complementary sets of Cys-containing homologs offered us a model system in which to analyze the origin and evolution of various selenoproteins. Although the majority of selenoprotein families have rare selenoproteins and widespread Cys-containing homologs (Additional data files 1 [Table S1] and 2 [Figure S2]), we found that several selenoproteins, including FdhA, SelW-like, and glycine reductase selenoproteins A (GrdA) and B (GrdB), have very few or even lack Cys-containing homologs in Sec-containing organisms (Additional data file 2 [Figure S3]). This observation suggests that Sec is the original form of these proteins. Moreover, by analyzing the phylogenies of 25 bacterial selenoprotein families, we detected more than twice as many Cys→Sec conversions as Sec→Cys events. In addition, the Cys→Sec conversions were detected in many thiol-based oxidoreductase families, suggesting that in most selenoprotein families there is a general trend toward Sec acquisition by replacement of catalytic redox-active Cys residues with Sec. It is possible that such replacements could be stabilized by vicinal residues in the active sites of these proteins. However, no such events were detected for FdhA, the most widely distributed and abundant selenoprotein family, as well as for SelD. We hypothesize that evolution of the Sec-decoding trait in most cases parallels the evolution of FdhA. Consistent with this idea, the genes for the Sec-decoding trait and FdhA are often in the same operon in Sec-decoding organisms, particularly those containing a single selenoprotein gene. Taken as a whole, these data suggest that acquisition of Sec-containing FdhA occurs via vertical or lateral inheritance of the Sec-decoding trait. SelD might be a second selenoprotein that helps to maintain the trait in organisms that lack FdhA. The requirement for FdhA or SelD to maintain the Sec-decoding trait and the scattered occurrence of other selenoproteins further illustrate a highly dynamic nature of Sec evolution.

Because new selenoproteins frequently evolve from their Cys-containing homologs, why do organisms have only a limited number of selenoproteins and why do so many organisms lack selenoproteins altogether? One hypothesis is that the Sec insertion trait is not stable, and evolution of new selenoproteins is balanced by selenoprotein loss in closely related organisms. To investigate the possibility of phylum-specific selenoprotein losses, we adopted an approach that relies on similarity between sister and relatively distant organisms. Similar methods have previously been used to analyze a general trend toward amino acid gain and loss in proteins [35]. Because the sister species selected for each selenoprotein-containing organism are closely related, the observed results directly reflect only about the past 30 million years of evolution. We found that all 38 selenoprotein loss events, including the six SelD losses that were accompanied by the loss of the entire Sec-decoding trait in sister genomes, occurred in the selenoprotein family-rich phyla *Firmicutes/Clostridia *and *Deltaproteobacteria*. Organisms in these phyla reflect a balanced pattern of ongoing selenoprotein origin and loss. The most plausible hypothesis to explain the loss of selenoproteins might relate to a universal, intrinsic, and long-term trend that emerged in both ancient and extant organisms. During this period, some ancient selenoprotein families might have been lost in most or all organisms, or some ancient organisms might have disappeared that contained ancient selenoproteins. Our hypothesis is consistent with the recently proposed 'balance hypothesis', which suggests that gene gain and loss in prokaryotes are balanced to keep prokaryotic genome size relatively constant [36]. However, the evolutionary forces modulating the balance are unclear.

**Figure 6:**
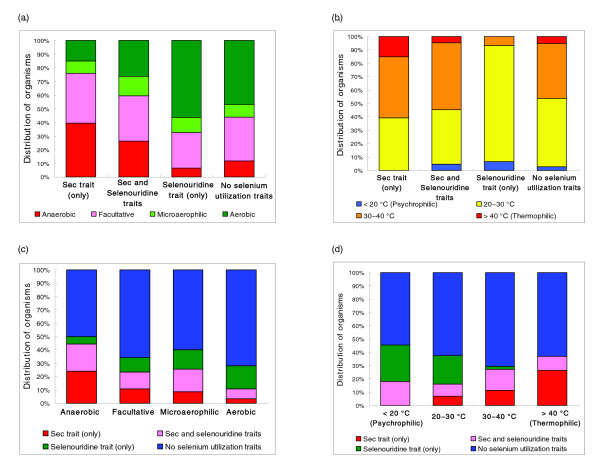
Relationship between selenium utilization traits and environmental factors (oxygen concentration and optimal growth temperature). Organisms were classified into four groups, including those containing the following: the Sec trait only, both Sec and selenouridine traits, selenouridine trait only, and no selenium utilization traits. **(a) **Distribution of organisms with different selenium utilization traits based on their requirement for oxygen. **(b) **Distribution of organisms with different selenium utilization traits based on their optimal growth temperature. **(c) **Distribution of organisms classified according to their oxygen requirement based on their selenium utilization traits. **(d) **Distribution of organisms classified according to their optimal growth temperature based on their selenium utilization traits.

To gain insight into the factors that influence maintenance/acquisition/loss of selenium utilization traits and Sec/Cys conversions, we analyzed environmental conditions (for example, habitat, oxygen requirement, optimal temperature, and optimal pH) and other factors (such as genome size and GC content) for all 349 bacteria for which completely or almost completely sequenced genomes are available, and compared those containing the Sec and/or selenouridine traits with those that do not. First, we found that the organisms possessing the Sec-decoding trait (especially those that have Sec but not selenouridine traits) favor anaerobic and hyperthermic conditions (Additional data file 1 [Tables S4 and S5] and Figure 6a, b). In contrast, organisms possessing the selenouridine trait (in the situations in which the Sec trait has been lost) favor aerobic environment and mesophilic conditions. Thus, decrease in oxygen concentration and increase in optimal growth temperature appeared to preserve or even stimulate the use of Sec (Figure 6c, d).

**Figure 7:**
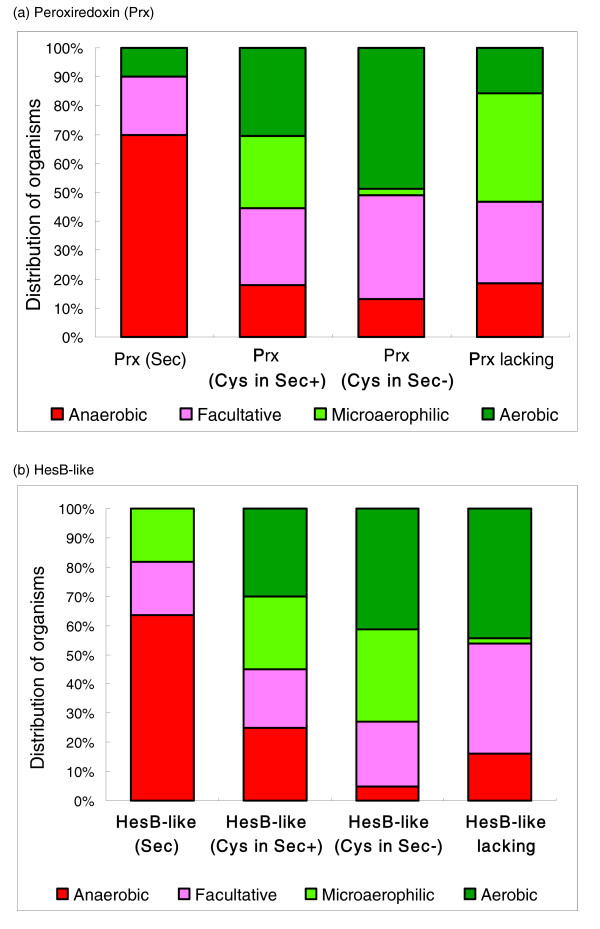
Relationships between two representative bacterial selenoprotein families and oxygen requirement of organisms containing these proteins. Organisms containing a member of a specific selenoprotein family (either Sec or Cys forms) were divided into four groups based on occurrence of the following: selenoproteins, Cys-containing homologs and the Sec trait, Cys-containing homologs and no Sec trait, and no representatives of this family. Distribution of organisms living under different oxygen conditions in each group is shown. **(a) **Peroxiredoxin (Prx). Peroxiredoxin homologs were identified by BLAST searches and included those containing the T/SxxU/C active site. **(b) **HesB-like protein. Sec^+^, organisms possessing the Sec-decoding trait; Sec^-^, organisms lacking the Sec-decoding trait.

Second, for various selenoprotein families, we examined distribution, based on several environmental factors, of organisms that have selenoproteins (the Sec form) and the Sec trait; Cys-containing homologs of selenoproteins (the Cys form) and the Sec trait; the Cys form and no Sec-decoding trait; and neither Sec nor Cys forms of selenoproteins and no Sec trait. For this analysis, we selected six selenoprotein families that have selenoproteins in at least 10 organisms and widespread Cys-containing homologs. For most of these selenoprotein families, a similar trend was found in which anaerobic conditions correlated with the presence of the Sec form (for instance, when species containing selenoproteins and the Sec trait were compared with those that had Cys-containing homologs and the Sec trait; see examples in Figure 7 and Additional data file 1 [Tables S6 and S7]). Our data again suggested that low oxygen level (anaerobic conditions) is the factor that promotes the use of Sec forms. It is possible that at high oxygen concentrations organisms could not tolerate the highly reactive Sec residue, which could be easily oxidized and could then support generation of reactive oxygen species. As a result, negative selection effects at the DNA level (either the loss of the whole Sec trait or Sec→Cys conversion) may be promoted under these conditions. Table 5 shows a summary of observed relationships between different environmental factors and conditions and selenium utilization traits. However, we did not observe a relationship between these factors and the number of selenoproteins (selenoproteome) in organisms, as well as between these factors and the presence/absence of different selenoprotein families. A future challenge would be to discover additional trends that influence selenium utilization in all three domains of life.

## Conclusion

We provided comprehensive phylogenetic analysis of selenoproteomes and Sec-decoding and selenouridine-utilizing traits in bacteria. Our data highlight a complex and highly dynamic evolutionary process for both selenium utilization traits and show, for the first time, HGT of selenoprotein genes between archaea and bacteria. The data also support the idea that FdhA is important for maintaining the Sec-decoding trait in bacteria. Multiple selenoprotein loss events identified in various selenoprotein families in selenoprotein-rich organisms suggest a dynamic balance between selenoprotein origin and loss during evolution. The primary events in selenoprotein evolution are Cys→Sec conversions and selenoprotein loss. Oxygen concentration and temperature appear to influence selenium utilization at the level of both Sec and selenouridine traits. Interestingly, although both of these traits utilize selenium, these environmental factors affected the traits in a contrasting manner.

## Materials and methods

### Sequences and resources

Both completely and incompletely sequenced bacterial genomes from the current Entrez Microbial Genome Project were used in this study (total of 349 species, 515 genomes; 1 April 2006). Information about environmental factors associated with these genomes was also retrieved from the NCBI database. We used *Escherichia coli *SelA, SelB, SelD, and YbbB sequences as queries to search for components of Sec-decoding and selenouridine traits. TBLASTN [37] was initially used to identify genes encoding homologs with a cut-off of E-value 0.01. Orthologs were then defined using the COG database (any two proteins from different lineages that belong to the same COG were considered orthologs) [38]. Furthermore, these orthologs must have shown more than 25% similarity in deduced amino acid sequence and less than 30% difference in length. Because of the large number of strains for some bacterial species, only one strain was selected from each species (for example, *Escherichia coli K12 *was used as a representative of *Escherichia coli*). *Shigella *species were not included because *Shigella *and *Escherichia coli *may belong to the same species based on DNA homology [39]. The presence of the Sec-decoding trait was verified by the additional requirement for the presence of at least one known selenoprotein gene. Seventy-five selenoprotein-containing bacteria were found and 84 *selA*, 75 *selB*, 127 *selD*, and 88 *ybbB *genes were identified.

Representative sequences derived from 24 bacterial selenoprotein families (excluding SelD) were used to search against the microbial genomic database for homologs with TBLASTN cutoff E-value 1.0. Sequences of both selenoproteins and Cys-containing homologs were retrieved and verified using COG and additional criteria discussed above. The presence of a putative Sec-encoding UGA codon and a downstream bacterial SECIS element were then analyzed using the bSECISearch program [28].

### Multiple sequence alignment, phylogenetic tree reconstruction and evaluation

To investigate the distribution of selenoprotein-containing organisms in various phyla, we adopted a phylogenetic tree recently developed by Ciccarelli and coworkers [23], which is based on concatenation of 31 orthologs occurring in 191 species with sequenced genomes.

To reconstruct phylogenetic trees of each component of selenium utilization traits and selenoprotein families, we used standard approaches. Sequences were aligned with CLUSTALW [40] and T-coffee [41] using default parameters. Ambiguous alignments in highly variable (gap-rich) regions were excluded. The resulting multiple alignments were then checked for conservation of functional residues and manually edited. Phylogenetic analyses were initially performed using PHYLIP programs [42]. Pairwise distance matrices were calculated by PROTDIST to estimate the expected amino acid replacements per position. Neighbor-joining trees were obtained with NEIGHBOR and the most parsimonious trees were determined with PROTPARS.

To evaluate the robustness of the trees, we also performed maximum likelihood analysis with PHYML [43] and Bayesian estimation of phylogeny with MrBayes [44] using different parameters (for example, transition matrices and evolutionary models). Moreover, considering that inclusion of gap characters could consistently improve support for nodes recovered by substitutions, we adopted an approach in which gap information was included as coded characters for better phylogenetic inference [45,46]. Alignable gaps of each protein were coded as binary (presence/absence) characters and added to the sequence matrix. This helped in the use of information on insertions/deletions during evolution and to resolve several additional nodes, most of which were apical on the phylogeny (ancient nodes) [45]. In total, 30 to 40 trees were analyzed for each protein. The final phylogenetic trees were then manually refined to reflect the most consistent topologies among these trees. Phylogenies of SECIS elements in different bacterial selenoprotein genes were analyzed with similar approaches.

In addition, we measured, for each pair of organisms, the correlation between the similarity of orthologous pairs and that of the 16S rRNA genes (control) to assess the general trend of the inferred trees. A method that had been successfully used to investigate gene essentiality in bacteria [47] was used. The indicator of protein similarity (or sequence divergence [47]) was defined based on both sequence similarity and length difference. The distance matrix of 16S rRNAs was calculated using DNADIST [48].

### Identification of conversion events between Sec-containing and Cys-containing proteins

In order to identify the possible conversion events between Sec- and Cys-containing forms of proteins in different selenoprotein families, we used the following logic, which is similar to that in previous analyses of an evolutionary trend of FdhA [28]. If a single Sec-containing sequence was clustered with a closely related Cys-containing sequence as well as with additional, more distantly related Cys-containing sequences, then we inferred that a Sec-containing protein evolved from a Cys-containing protein (Cys→Sec conversion). Likewise, if a single Cys-containing sequence clustered with both closely related and more distantly related Sec-containing sequences, then Sec→Cys conversion was inferred. If several Sec-containing sequences from evolutionarily close organisms clustered with Cys-containing sequences and additional Cys-containing homologs were at the root of the tree, then only one conversion event was considered.

### Verification of selenoprotein loss event in sister species

The authenticity of phylum-specific selenoprotein gene loss events was verified by considering the distribution of Sec/Cys-containing orthologous proteins in both evolutionarily close genomes (sister species) and relatively distant genomes.

First, for each focused selenoprotein-containing organism, we selected two or three sister species and three or four relatively distant species in the same phylum based on the newly developed phylogenetic tree of life [23]. Only complete or almost complete genomes were considered for this analysis in order to avoid the possibility that the homolog has not been sequenced. If a phylum contained too many sequenced genomes, then we divided it into several taxa subgroups and selected relatively distant species from different subgroups. For example, for *Escherichia coli*, which is a selenoprotein-containing organism in *Gammaproteobacteria/Enterobacteriales*, we defined *Salmonella enterica, Yersinia pestis KIM*, and *Photorhabdus luminescens *as sister species, as well as defining *Vibrio cholerae *(*Gammaproteobacteria/Vibrionales*), *Haemophilus influenzae *(*Gammaproteobacteria/Pasteurellales*), *Shewanella oneidensis *(*Gammaproteobacteria/Alteromonadales*), and *Pseudomonas aeruginosa *(*Gammaproteobacteria/Pseudomonadales*) as relatively distant species. A complete list of sister and relatively distant species for each selenoprotein-containing organism is shown in Additional data file 1 (Table S2).

Second, for each selenoprotein family in focused species, we defined *O*_*sis *_as the occurrence of Sec/Cys-containing homologs in sister species and *O*_*dis *_as that in relatively distant species. The following evolutionary situations were considered. First, if *O*_*sis *_≤ 1 and *O*_*dis *_≥ 2, or if *O*_*sis *_≤ 1 and *O*_*dis *_≤ 1 and the distant homolog is most homologous to the query selenoprotein compared with homologs in other phyla (suggesting a vertical descent but not an unrelated HGT), then the case was taken as an indication that the selenoprotein family being analyzed may be subject to selenoprotein loss (*O*_*sis *_is 0) or the loss in progress (*O*_*sis *_is 1) in the current phylum. Second, if *O*_*sis *_> 1 and *O*_*dis *_> 2, then the case was taken as an indication that this selenoprotein family is functionally conserved and widespread in the current phylum. In this case, the loss of selenoproteins was not detected. Finally, if neither of the first two situations could be satisfied, then we could not assign a clear evolutionary pattern. For example, if neither sister nor relatively distant species contained Sec/Cys-containing sequences (in the case of *O*_*sis *_< 1 and *O*_*dis *_< 2), or Sec/Cys-containing sequences were abundant in sister species but rare or absent in distantly related species (*O*_*sis *_> 1 and *O*_*dis *_< 2), then either selenoprotein loss or gain could be possible.

This method allowed us to identify phylum-specific selenoprotein loss events with high degree of certainty. Furthermore, because it is also possible for distant species to acquire homologous genes by HGT from other phyla, the constraints *O*_*sis *_= 1 and *O*_*dis *_= 1, as well as an additional requirement that distant homolog is most homologous to the query selenoprotein compared with homologs in other phyla, allowed us to determine whether phylum-specific selenoprotein loss event had really happened. Therefore, the orthology was confirmed by such criterion if only one homolog was available in distant organisms.

## Additional data files

The following additional data files are available with the online version of this paper. Additional data file 1 contains seven supplemental tables. Additional data file 2 includes three supplemental figures. Additional data file 3 provides sequences of components of selenium-utilization traits (SelA, SelB, SelD, and YbbB) in bacteria. Additional data file 4 provides sequences of all 285 bacterial selenoproteins.

## Supplementary Material

Additional data file 1 Table S1 contains data on distribution of selenoproteins and their Cys-containing homologs in different organisms. Table S2 includes the complete list of sister and more distant species of selenoprotein-containing bacteria. Table S3 contains information about selenoprotein loss events identified in 25 bacterial selenoprotein families. Tables S4 and S5 show the distribution of organisms, which have or lack selenium utilization traits as analyzed by considering different environmental factors. Tables S6 and S7 show the distribution of organisms, which have or lack Sec-/Cys-containing form of peroxiredoxin, and HesB-like protein as analyzed by considering oxygen requirement. Click here for file

Additional data file 2 Figure S1 shows phylograms of SelA, SelB, SelD, and YbbB sequences. Figures S2 and S3 show the distribution of selenoproteins and their Cys-containing homologs in different organisms. Click here for file

Additional data file 3 Sequences of components of selenium utilization traits (SelA, SelB, SelD and YbbB) in bacteria. Sec is represented by U Click here for file

Additional data file 4 Sequences of all 285 selenoproteins (25 families) in bacteria. Sec is represented by U Click here for file

## Figures and Tables

**Table 1:** Distribution and Sec evolutionary trends of 25 bacterial selenoprotein families

Selenoprotein family	Number of selenoproteins	Sec/Cys conversion events		Selenoprotein loss events
			
		Sec→Cys	Cys→Sec	
Formate dehydrogenase alpha subunit (FdhA)	103	7	-	2
Selenophosphate synthetase (SelD)	38	3	-	6
Coenzyme F420-reducing hydrogenase delta subunit (FrhD)^a^	19	3	3	5
Heterodisulfide reductase, subunit A (HdrA)^a^	16	-	2	4
Peroxiredoxin (Prx)^a^	12	-	5	-
HesB-like	11	2	-	3
Glycine reductase selenoprotein A (GrdA)	11	-	-	4
Glycine reductase selenoprotein B (GrdB)^a^	11	-	-	6
SelW-like^a^	10	-	-	3
Prx-like thiol:disulfide oxidoreductase^a^	8	-	3	-
Thioredoxin (Trx)^a^	7	-	-	-
Coenzyme F420-reducing hydrogenase α subunit (FrhA)^a^	6	-	2	-
Fe-S oxidoreductase (GlpC)	5	-	2	-
Proline reductase (PR)^a^	5	-	-	-
DsbA-like^a^	4	-	1	1
Glutaredoxin (Grx)^a^	3	-	3	-
Thiol:disulfide interchange protein^a^	3	-	1	-
AhpD-like (COG2128)^a^	2	-	2	-
ArsC-like^a^	2	-	1	2
DsbG-like^a^	2	-	2	-
Distant AhpD homolog^a^	2	-	1	-
Homolog of AhpF, amino-terminal domain^a^	2	-	2	1
DsrE-like^a^	1	-	1	-
NADH oxidase	1	-	1	1
Glutathione peroxidase (GPx)^a^	1	-	1	-

Total	285	15	33	38

^a^Homologs of thiol-based oxidoreductases.

**Table 2:** Distribution of 25 selenoprotein families in bacterial phyla/branches

Phyla	Number of selenoprotein families	Number of selenoproteins
*Deltaproteobacteria*	22	121
*Firmicutes/Clostridia*	16	58
*Actinobacteria*	12	19
*Spirochaetes*	5	6
*Chloroflexi*	3	3
Acidobacteria	3	4
*Firmicutes*/*Bacillales*	3	3
*Epsilonproteobacteria*	3	18
*Gammaproteobacteria/Vibrionales*	3	6
*Aquificae*	2	2
*Gammaproteobacteria/Pasteurellales*	2	9
*Alphaproteobacteria*	1	3
*Betaproteobacteria*	1	10
*Gammaproteobacteria *(other than listed)	1	23

Total	25	285

**Table 3:** Selenoproteomes and environmental conditions of 18 selenoprotein-rich organisms

Phyla/organisms	Number of selenoproteins	Selenoproteins (number)	Aerobic/anaerobic	Temperature (°C)
*Deltaproteobacteria*				
*Syntrophobacter fumaroxidans*	31	SelD, FdhA (6), FrhA (3), FrhD (8), HdrA (7), GlpC (3), peroxiredoxin, HesB-like, MsrA	Anaerobic	20-25
*Syntrophus aciditrophicus*	19	SelD, FdhA (4), FrhD (4), HdrA (4), peroxiredoxin, GrdA, GrdB, Prx-like thiol:disulfide oxidoreductase, thiol:disulfide interchange protein, HesB-like	Anaerobic	20-25
*Desulfotalea psychrophila*	12	SelD, FdhA (4), GlpC, Prx-like thiol:disulfide oxidoreductase, SelW-like, FrhA, FrhD, HdrA, ArsC-like	Anaerobic	7-10
*Anaeromyxobacter dehalogenans*	11	FdhA (3), SelD, peroxiredoxin (3), proline reductase, thioredoxin (2), DsbA-like	Anaerobic	30
*Desulfovibrio vulgaris*	8	SelD, FdhA (3), DsrE-like, GlpC, HesB-like, FrhA	Anaerobic	25-40
*Geobacter metallireducens*	8	SelD, FdhA, Prx-like thiol:disulfide oxidoreductase, thioredoxin, FrhD, peroxiredoxin, thiol:disulfide interchange protein, NADH oxidase	Anaerobic	25-30
*Geobacter sulfurreducens*	8	SelD, FdhA, Prx-like thiol:disulfide oxidoreductase, thioredoxin, distant AhpD homolog, glutaredoxin, HesB-like, SelW-like	Anaerobic	30-35
*Geobacter uraniumreducens*	8	FdhA (2), SelD, Prx-like, thioredoxin, proline reductase, thiol:disulfide interchange protein, distant AhpD homolog	Anaerobic	30-35
*Desulfovibrio desulfuricans*	7	SelD, FdhA (3), FrhA, HesB-like, DSBA-like	Anaerobic	25-40
*Desulfuromonas acetoxidans*	6	SelD, GrdA (2), GrdB, HesB-like, distant ArsC homolog	Anaerobic	25-30
*Firmicutes/Clostridia*				
*Alkaliphilus metalliredigenes*	11	FdhA, peroxiredoxin (2), GrdA, GrdB, proline reductase, HesB-like, glutaredoxin (2), SelW-like, AhpD-like (COG2128)	Facultative	30
*Syntrophomonas wolfei*	10	SelD, FdhA (5), FrhD, HdrA, peroxiredoxin, distant Prx-like thiol:disulfide oxidoreductase	Anaerobic	20-25
*Carboxydothermus hydrogenoformans*	9	SelD, FdhA (2), GrdA, GrdB, homolog of AhpF N-terminal domain, FrhD, thioredoxin, HdrA	Anaerobic	78
*Desulfotomaculum reducens*	8	SelD, FdhA (2), FrhD (2), HdrA, SelW-like, DsbA-like	Anaerobic	20-25
*Clostridium difficile*	6	SelD, FdhA, GrdA, GrdB (2), proline reductase	Anaerobic	25-40
*Moorella thermoacetica*	6	SelD, FdhA (2), HdrA, FrhD, glutaredoxin	Anaerobic	58
*Actinobacteria*				
*Symbiobacterium thermophilum*	12	FdhA (3), SelD, GrdA, GrdB, HesB-like, AhpF N-terminal domain, peroxiredoxin, SelW-like, DsbG-like	Microaerophile	60
*Spirochaetes*				
*Treponema denticola*	6	SelD, Gpx, GrdA, GrdB (2), thioredoxin	Anaerobic	30-42

**Table 4:** Events of selenoprotein loss identified in different bacterial phyla

Phylum/organism	Number of selenoproteins	Selenoprotein families lost in sister organisms
*Deltaproteobacteria*		
*Syntrophus aciditrophicus*	19	GrdB
*Desulfotalea psychrophila*	12	SelW-like, ArsC-like
*Geobacter metallireducens*	8	NADH oxidase
*Geobacter sulfurreducens*	8	HesB-like, SelW-like
*Desulfuromonas acetoxidans*	6	GrdB, ArsC-like
*Firmicutes/Clostridia*		
*Alkaliphilus metalliredigenes*	11	GrdA, GrdB
*Syntrophomonas wolfei*	10	FrhD, HdrA
*Carboxydothermus hydrogenoformans*	9	GrdA, GrdB, homolog of AhpF N-terminal domain, FrhD, HdrA
*Desulfotomaculum reducens*	8	FrhD, HdrA, DsbA-like
*Clostridium difficile*	6	SelD, FdhA, GrdA, GrdB
*Moorella thermoacetica*	6	SelD, FdhA, HdrA, FrhD
*Thermoanaerobacter tengcongensis*	3	SelD, GrdA, GrdB
*Desulfitobacterium hafniense*	3	HesB-like
*Clostridium perfringens*	2	SelD
*Actinobacteria*		
*Symbiobacterium thermophilum*	12	SelD, HesB-like, SelW-like
*Rubrobacter xylanophilus*	5	SelD

**Table 5:** General trends and correlations between changes in environmental factors, occurrence of selenium utilization traits, and occurrence of selenoproteins and their Cys-containing homologs in bacteria

Environmental factor		Sec-decoding trait	Selenouridine trait	Selenoproteins^a^	Cys-containing homologs^a^
Oxygen concentration	**↑**	**↓**	**↑**	**↓**	**↑**
	**↓**	**↑**	**↓**	**↑**	**↓**
Temperature	**↑**	**↑**	**↓**	ND	ND
	**↓**	**↓**	**↑**	ND	ND

^a^As exemplified by Prx and HesB-like proteins. ND, not determined.
