# Supplementary material for: Dynamic evolution of selenocysteine utilization in bacteria: a balance between selenoprotein loss and evolution of selenocysteine from redox active cysteine residues
Source: Genome Biol. 2006 Oct 20;7(10):R94. doi: 10.1186/gb-2006-7-10-r94 (PMC1794560; doi:10.1186/gb-2006-7-10-r94)
Supplement: Additional data file 1 — Table S1 contains data on distribution of selenoproteins and their Cys-containing homologs in different organisms. Table S2 includes the complete list of sister and more distant species of selenoprotein-containing bacteria. Table S3 contains information about selenoprotein loss events identified in 25 bacterial selenoprotein families. Tables S4 and S5 show the distribution of organisms, which have or lack selenium utilization traits as analyzed by considering different environmental factors. Tables S6 and S7 show the distribution of organisms, which have or lack Sec-/Cys-containing form of peroxiredoxin, and HesB-like protein as analyzed by considering oxygen requirement. [file gb-2006-7-10-r94-S1.pdf]

**Table S1. Distribution of Sec-/Cys-containing sequences in different organisms**

| Selenoprotein family | Number of organisms containing both Sec form and Sec trait | Number of organisms containing only Cys form and Sec trait | Number of organisms containing Cys form but no Sec trait |
|---|---|---|---|
| FdhA | 67 | 6 | 126 |
| SelD | 38 | 37 | 46 |
| FrhD | 10 | 5 | 9 |
| HdrA | 7 | 11 | 29 |
| Peroxiredoxin | 10 | 56 | 251 |
| HesB-like | 11 | 20 | 63 |
| GrdA | 10 | 0 | 0 |
| GrdB | 10 | 1 | 5 |
| SelW-like | 10 | 0 | 13 |
| Prx-like | 7 | 11 | 26 |
| Thioredoxin | 7 | 61 | 278 |
| FrhA | 4 | 12 | 24 |
| GlpC | 3 | 8 | 6 |
| Proline reductase | 5 | 1 | 7 |
| DsbA-like | 4 | 58 | 169 |
| Glutaredoxin | 3 | 45 | 237 |
| Thiol:disulfide interchange protein | 3 | 46 | 114 |
| AhpD-like | 2 | 59 | 228 |
| ArsC-like | 2 | 1 | 4 |
| DsbG-like | 2 | 10 | 31 |
| Distant AhpD homolog | 2 | 23 | 64 |
| Homolog of AhpF, N-terminal domain | 2 | 35 | 88 |
| DsrE-like | 1 | 0 | 4 |
| NADH oxidase | 1 | 59 | 231 |
| GPx | 1 | 63 | 244 |

## Table S2. Complete list of sister and more distant species of selenoprotein-containing bacteria

| Phylum | Organism | Sister organisms | | | Distant organisms | | | |
|---|---|---|---|---|---|---|---|---|
| | | 1 | 2 | 3 | 1 | 2 | 3 | 4 |
| **Actinobacteria** | *Symbiobacterium thermophilum* | *Rubrobacter xylanophilus* | *Bifidobacterium longum* | *Tropheryma whipplei* | *Kineococcus radiotolerans* | *Mycobacterium avium* | *Brevibacterium linens* | *Corynebacterium diphtheriae* |
| | *Rubrobacter xylanophilus* | *Symbiobacterium thermophilum* | *Bifidobacterium longum* | *Tropheryma whipplei* | *Kineococcus radiotolerans* | *Mycobacterium avium* | *Brevibacterium linens* | *Corynebacterium diphtheriae* |
| | *Brevibacterium linens* | *Tropheryma whipplei* | *Leifsonia xyli* | *Arthrobacter sp.* | *Rubrobacter xylanophilus* | *Kineococcus radiotolerans* | *Mycobacterium avium* | *Corynebacterium diphtheriae* |
| | *Mycobacterium avium* | *Mycobacterium bovis* | *Mycobacterium leprae* | *Mycobacterium sp.* | *Brevibacterium linens* | *Kineococcus radiotolerans* | *Rubrobacter xylanophilus* | *Symbiobacterium thermophilum* |
| | *Kineococcus radiotolerans* | *Frankia sp.* | *Acidothermus cellulolyticus* | - | *Mycobacterium avium* | *Brevibacterium linens* | *Symbiobacterium thermophilum* | *Corynebacterium diphtheriae* |
| **Acidobacteria** | *Solibacter usitatus* | - | - | - | *Acidobacteria bacterium* | - | - | - |
| **Aquificae** | *Aquifex aeolicus* | - | - | - | - | - | - | - |
| **Chloroflexi** | *Chloroflexus aurantiacus* | *Chloroflexus aggregans* | *Roseiflexus sp.* | *Heliothrix oregonensis* | *Dehalococcoides ethenogenes* | *Thermomicrobium roseum* | *Candidatus Chlorothrix halophila* | - |
| **Spirochaetes** | *Treponema denticola* | *Treponema pallidum* | *Borrelia garinii* | *Borrelia burgdorferi* | *Leptospira interrogans* | - | - | - |
| ***Deltaproteobacteria*** | *Syntrophobacter fumaroxidans* | *Syntrophus aciditrophicus* | *Syntrophus buswellii* | - | *Desulfotalea psychrophila* | *Desulfovibrio vulgaris* | *Geobacter metallireducens* | *Desulfuromonas acetoxidans* |
| | *Desulfotalea psychrophila* | *Desulfobacterium autotrophicum* | *Nitrospina gracilis* | - | *Geobacter sulfurreducens* | *Desulfuromonas acetoxidans* | *Desulfovibrio vulgaris* | *Anaeromyxobacter dehalogenans* |
| | *Desulfovibrio desulfuricans* | *Desulfovibrio vulgaris* | *Lawsonia intracellularis* | - | *Geobacter sulfurreducens* | *Desulfotalea psychrophila* | *Desulfuromonas acetoxidans* | *Anaeromyxobacter dehalogenans* |
| | *Desulfovibrio vulgaris* | *Desulfovibrio desulfuricans* | *Lawsonia intracellularis* | - | *Geobacter sulfurreducens* | *Desulfotalea psychrophila* | *Desulfuromonas acetoxidans* | *Anaeromyxobacter dehalogenans* |
| | *Syntrophus aciditrophicus* | *Syntrophus buswellii* | *Syntrophobacter fumaroxidans* | - | *Desulfuromonas acetoxidans* | *Desulfotalea psychrophila* | *Desulfovibrio vulgaris* | *Geobacter sulfurreducens* |
| | *Pelobacter carbinolicus* | *Pelobacter propionicus* | *Geobacter sulfurreducens* | *Geobacter metallireducens* | *Desulfotalea psychrophila* | *Desulfovibrio vulgaris* | *Syntrophus aciditrophicus* | *Anaeromyxobacter dehalogenans* |
| | *Geobacter metallireducens* | *Geobacter sulfurreducens* | *Geobacter uraniumreducens* | *Pelobacter carbinolicus* | *Desulfotalea psychrophila* | *Desulfovibrio vulgaris* | *Syntrophus aciditrophicus* | *Desulfuromonas acetoxidans* |
| | *Geobacter sulfurreducens* | *Geobacter metallireducens* | *Geobacter uraniumreducens* | *Pelobacter carbinolicus* | *Desulfotalea psychrophila* | *Desulfovibrio vulgaris* | *Syntrophus aciditrophicus* | *Desulfuromonas acetoxidans* |
| | *Geobacter uraniumreducens* | *Geobacter sulfurreducens* | *Geobacter metallireducens* | *Pelobacter carbinolicus* | *Desulfotalea psychrophila* | *Desulfovibrio vulgaris* | *Syntrophus aciditrophicus* | *Desulfuromonas acetoxidans* |
| | *Desulfuromonas acetoxidans* | *Geobacter metallireducens* | *Geobacter sulfurreducens* | *Pelobacter carbinolicus* | *Desulfotalea psychrophila* | *Syntrophus aciditrophicus* | *Anaeromyxobacter dehalogenans* | *Desulfovibrio vulgaris* |
| | *Anaeromyxobacter dehalogenans* | *Myxococcus xanthus* | - | - | *Desulfotalea psychrophila* | *Syntrophus aciditrophicus* | *Desulfovibrio vulgaris* | *Geobacter metallireducens* |
| ***Firmicutes*/*Bacillales*** | *Bacillus sp.* | *Bacillus anthracis* | *Bacillus clausii* | *Bacillus thuringiensis* | *Listeria innocua* | *Oceanobacillus iheyensis* | *Staphylococcus aureus* | *Geobacillus kaustophilus* |
| **Firmicutes/ Clostridia** | *Moorella thermoacetica* | *Thermoanaerobacter ethanolicus* | *Thermoanaerobacter tengcongensis* | *Ammonifex degensii* | *Carboxydothermus hydrogenoformans* | *Desulfitobacterium hafniense* | *Clostridium difficile* | *Desulfotomaculum reducens* |
| | *Syntrophomonas wolfei* | *Caldicellulosiruptor saccharolyticus* | *Anaerobranca gottschalkii* | - | *Moorella thermoacetica* | *Carboxydothermus hydrogenoformans* | *Thermoanaerobacter tengcongensis* | *Desulfotomaculum reducens* |

| | | | | | | | | |
|---|---|---|---|---|---|---|---|---|
| | *Desulfotomaculum reducens* | *Carboxydothermus hydrogenoformans* | *Desulfitobacterium hafniense* | - | *Moorella thermoacetica* | *Syntrophomonas wolfei* | *Clostridium difficile* | *Thermoanaerobacter tengcongensis* |
| | *Carboxydothermus hydrogenoformans* | *Desulfotomaculum reducens* | *Desulfitobacterium hafniense* | - | *Moorella thermoacetica* | *Syntrophomonas wolfei* | *Clostridium difficile* | *Thermoanaerobacter tengcongensis* |
| | *Thermoanaerobacter tengcongensis* | *Moorella thermoacetica* | *Thermoanaerobacter ethanolicus* | *Ammonifex degensii* | *Carboxydothermus hydrogenoformans* | *Desulfitobacterium hafniense* | *Clostridium difficile* | *Desulfotomaculum reducens* |
| | *Clostridium perfringens* | *Clostridium difficile* | *Clostridium tetani* | *Clostridium botulinum* | *Thermoanaerobacter tengcongensis* | *Moorella thermoacetica* | *Desulfitobacterium hafniense* | *Carboxydothermus hydrogenoformans* |
| | *Clostridium difficile* | *Clostridium perfringens* | *Clostridium tetani* | *Clostridium botulinum* | *Thermoanaerobacter tengcongensis* | *Moorella thermoacetica* | *Desulfitobacterium hafniense* | *Carboxydothermus hydrogenoformans* |
| | *Alkaliphilus metalliredigenes* | *Clostridium difficile* | *Clostridium perfringens* | *Clostridium tetani* | *Carboxydothermus hydrogenoformans* | *Thermoanaerobacter tengcongensis* | *Desulfotomaculum reducens* | *Moorella thermoacetica* |
| | *Desulfitobacterium hafniense* | *Carboxydothermus hydrogenoformans* | *Desulfotomaculum reducens* | - | *Clostridium perfringens* | *Alkaliphilus metalliredigenes* | *Thermoanaerobacter tengcongensis* | *Moorella thermoacetica* |
| ***Alphaproteobacteria*** | *Sulfitobacter sp.* | *Jannaschia sp.* | *Paracoccus denitrificans* | *Rhodobacter sphaeroides* | *Xanthobacter autotrophicus* | *Sinorhizobium meliloti* | *Silicibacter sp.* | *Rickettsia bellii* |
| | *Paracoccus denitrificans* | *Sulfitobacter sp.* | *Jannaschia sp.* | *Rhodobacter sphaeroides* | *Xanthobacter autotrophicus* | *Sinorhizobium meliloti* | *Silicibacter sp.* | *Rickettsia bellii* |
| | *Xanthobacter autotrophicus* | *Brucella suis* | *Bartonella quintana* | *Sinorhizobium meliloti* | *Paracoccus denitrificans* | *Silicibacter sp.* | *Rickettsia bellii* | *Acidiphilium cryptum* |
| | *Sinorhizobium meliloti pSymA* | *Rhizobium etli* | *Agrobacterium tumefaciens* | *Xanthobacter autotrophicus* | *Paracoccus denitrificans* | *Silicibacter sp.* | *Rickettsia bellii* | *Acidiphilium cryptum* |
| ***Betaproteobacteria*** | *Dechloromonas aromatica* | *Azoarcus sp.* | - | - | *Bordetella bronchiseptica* | *Burkholderia pseudomallei* | *Chromobacterium violaceum* | *Thiobacillus denitrificans* |
| | *Burkholderia fungorum* | *Burkholderia mallei* | *Burkholderia pseudomallei* | *Burkholderia vietnamiensis* | *Dechloromonas aromatica* | *Bordetella bronchiseptica* | *Chromobacterium violaceum* | *Thiobacillus denitrificans* |
| | *Burkholderia thailandensis* | *Burkholderia mallei* | *Burkholderia pseudomallei* | *Burkholderia ambifaria* | *Dechloromonas aromatica* | *Bordetella bronchiseptica* | *Chromobacterium violaceum* | *Thiobacillus denitrificans* |
| | *Burkholderia pseudomallei* | *Burkholderia mallei* | *Burkholderia ambifaria* | *Burkholderia fungorum* | *Dechloromonas aromatica* | *Bordetella bronchiseptica* | *Chromobacterium violaceum* | *Thiobacillus denitrificans* |
| | *Burkholderia mallei* | *Burkholderia pseudomallei* | *Burkholderia vietnamiensis* | *Burkholderia cenocepacia* | *Dechloromonas aromatica* | *Bordetella bronchiseptica* | *Chromobacterium violaceum* | *Thiobacillus denitrificans* |
| | *Burkholderia ambifaria* | *Burkholderia mallei* | *Burkholderia pseudomallei* | *Burkholderia cenocepacia* | *Dechloromonas aromatica* | *Bordetella bronchiseptica* | *Chromobacterium violaceum* | *Thiobacillus denitrificans* |
| | *Burkholderia vietnamiensis* | *Burkholderia mallei* | *Burkholderia pseudomallei* | *Burkholderia dolosa* | *Dechloromonas aromatica* | *Bordetella bronchiseptica* | *Chromobacterium violaceum* | *Thiobacillus denitrificans* |
| | *Burkholderia dolosa* | *Burkholderia mallei* | *Burkholderia ambifaria* | *Burkholderia fungorum* | *Dechloromonas aromatica* | *Bordetella bronchiseptica* | *Chromobacterium violaceum* | *Thiobacillus denitrificans* |
| | *Burkholderia cenocepacia* | *Burkholderia pseudomallei* | *Burkholderia dolosa* | *Burkholderia mallei* | *Dechloromonas aromatica* | *Bordetella bronchiseptica* | *Chromobacterium violaceum* | *Thiobacillus denitrificans* |
| | *Burkholderia sp.* | *Burkholderia mallei* | *Burkholderia pseudomallei* | *Burkholderia cenocepacia* | *Dechloromonas aromatica* | *Bordetella bronchiseptica* | *Chromobacterium violaceum* | *Thiobacillus denitrificans* |
| ***Epsilonproteobacteria*** | *Wolinella succinogenes* | *Helicobacter hepaticus* | *Helicobacter pylori* | *Thiomicrospira denitrificans* | *Campylobacter jejuni* | *Campylobacter upsaliensis* | *Nautilia sp.* | - |
| | *Helicobacter hepaticus* | *Helicobacter pylori* | *Wolinella succinogenes* | *Thiomicrospira denitrificans* | *Campylobacter jejuni* | *Campylobacter upsaliensis* | *Nautilia sp.* | - |
| | *Thiomicrospira denitrificans* | *Wolinella succinogenes* | *Helicobacter hepaticus* | *Helicobacter pylori* | *Campylobacter coli* | *Campylobacter jejuni* | *Nautilia sp.* | - |
| | *Campylobacter fetus* | *Campylobacter jejuni* | *Campylobacter coli* | *Campylobacter upsaliensis* | *Wolinella succinogenes* | *Helicobacter hepaticus* | *Nautilia sp.* | *Thiomicrospira denitrificans* |

| | | | | | | | | |
|---|---|---|---|---|---|---|---|---|
| | *Campylobacter lari* | *Campylobacter jejuni* | *Campylobacter coli* | *Campylobacter upsaliensis* | *Wolinella succinogenes* | *Helicobacter hepaticus* | *Nautilia sp.* | *Thiomicrospira denitrificans* |
| | *Campylobacter upsaliensis* | *Campylobacter jejuni* | *Campylobacter coli* | *Campylobacter fetus* | *Wolinella succinogenes* | *Helicobacter hepaticus* | *Nautilia sp.* | *Thiomicrospira denitrificans* |
| | *Campylobacter jejuni* | *Campylobacter coli* | *Campylobacter upsaliensis* | *Campylobacter upsaliensis* | *Wolinella succinogenes* | *Helicobacter hepaticus* | *Nautilia sp.* | *Thiomicrospira denitrificans* |
| | *Campylobacter coli* | *Campylobacter jejuni* | *Campylobacter upsaliensis* | *Campylobacter coli* | *Wolinella succinogenes* | *Helicobacter hepaticus* | *Nautilia sp.* | *Thiomicrospira denitrificans* |
| ***Gammaproteobacteria*** | *Photobacterium sp.* | *Photobacterium profundum* | *Vibrio cholerae* | *Vibrio vulnificus* | *Escherichia coli* | *Shewanella oneidensis* | *Pseudomonas aeruginosa* | *Haemophilus influenzae* |
| | *Photobacterium profundum* | *Photobacterium sp.* | *Vibrio cholerae* | *Vibrio vulnificus* | *Escherichia coli* | *Shewanella oneidensis* | *Pseudomonas aeruginosa* | *Haemophilus influenzae* |
| | *Vibrio angustum* | *Vibrio cholerae* | *Vibrio vulnificus* | *Photobacterium profundum* | *Escherichia coli* | *Shewanella oneidensis* | *Pseudomonas aeruginosa* | *Haemophilus influenzae* |
| | *Pseudomonas aeruginosa* | *Pseudomonas fluorescens* | *Pseudomonas putida* | *Pseudomonas syringae* | *Escherichia coli* | *Shewanella oneidensis* | *Haemophilus influenzae* | *Vibrio cholerae* |
| | *Pseudomonas fluorescens* | *Pseudomonas aeruginosa* | *Pseudomonas putida* | *Pseudomonas syringae* | *Escherichia coli* | *Shewanella oneidensis* | *Haemophilus influenzae* | *Vibrio cholerae* |
| | *Pseudomonas putida* | *Pseudomonas aeruginosa* | *Pseudomonas fluorescens* | *Pseudomonas syringae* | *Escherichia coli* | *Shewanella oneidensis* | *Haemophilus influenzae* | *Vibrio cholerae* |
| | *Shewanella sp.* | *Shewanella oneidensis* | *Shewanella denitrificans* | *Colwellia psychrerythraea* | *Haemophilus influenzae* | *Escherichia coli* | *Vibrio cholerae* | *Pseudomonas aeruginosa* |
| | *Shewanella oneidensis* | *Shewanella denitrificans* | *Shewanella sp.* | *Colwellia psychrerythraea* | *Haemophilus influenzae* | *Escherichia coli* | *Vibrio cholerae* | *Pseudomonas aeruginosa* |
| | *Actinobacillus pleuropneumoniae* | *Actinobacillus succinogenes* | *Haemophilus influenzae* | *Mannheimia succiniciproducens* | *Escherichia coli* | *Vibrio cholerae* | *Shewanella oneidensis* | *Pseudomonas putida* |
| | *Haemophilus ducreyi* | *Haemophilus influenzae* | *Mannheimia succiniciproducens* | *Pasteurella multocida* | *Escherichia coli* | *Vibrio cholerae* | *Shewanella oneidensis* | *Pseudomonas putida* |
| | *Haemophilus influenzae* | *Mannheimia succiniciproducens* | *Pasteurella multocida* | *Haemophilus ducreyi* | *Escherichia coli* | *Vibrio cholerae* | *Shewanella oneidensis* | *Pseudomonas putida* |
| | *Pasteurella multocida* | *Mannheimia succiniciproducens* | *Haemophilus influenzae* | *Actinobacillus succinogenes* | *Escherichia coli* | *Vibrio cholerae* | *Shewanella oneidensis* | *Pseudomonas putida* |
| | *Actinobacillus succinogenes* | *Actinobacillus pleuropneumoniae* | *Haemophilus influenzae* | *Pasteurella multocida* | *Escherichia coli* | *Vibrio cholerae* | *Shewanella oneidensis* | *Pseudomonas putida* |
| | *Mannheimia succiniciproducens* | *Haemophilus influenzae* | *Actinobacillus pleuropneumoniae* | *Pasteurella multocida* | *Escherichia coli* | *Vibrio cholerae* | *Shewanella oneidensis* | *Pseudomonas putida* |
| | *Photorhabdus luminescens* | *Escherichia coli* | *Yersinia pestis KIM* | *Salmonella enterica* | *Vibrio cholerae* | *Haemophilus influenzae* | *Shewanella oneidensis* | *Pseudomonas putida* |
| | *Yersinia pseudotuberculosis* | *Escherichia coli* | *Yersinia pestis KIM* | *Photorhabdus luminescens* | *Vibrio cholerae* | *Haemophilus influenzae* | *Shewanella oneidensis* | *Pseudomonas aeruginosa* |
| | *Yersinia pestis KIM* | *Escherichia coli* | *Yersinia pseudotuberculosis* | *Photorhabdus luminescens* | *Vibrio cholerae* | *Haemophilus influenzae* | *Shewanella oneidensis* | *Pseudomonas aeruginosa* |
| | *Yersinia intermedia* | *Escherichia coli* | *Yersinia pestis KIM* | *Photorhabdus luminescens* | *Vibrio cholerae* | *Haemophilus influenzae* | *Shewanella oneidensis* | *Pseudomonas aeruginosa* |
| | *Yersinia frederiksenii* | *Escherichia coli* | *Yersinia pseudotuberculosis* | *Photorhabdus luminescens* | *Vibrio cholerae* | *Haemophilus influenzae* | *Shewanella oneidensis* | *Pseudomonas aeruginosa* |
| | *Yersinia mollaretii* | *Escherichia coli* | *Yersinia pseudotuberculosis* | *Photorhabdus luminescens* | *Vibrio cholerae* | *Haemophilus influenzae* | *Shewanella oneidensis* | *Pseudomonas aeruginosa* |
| | *Yersinia bercovieri* | *Escherichia coli* | *Yersinia pseudotuberculosis* | *Photorhabdus luminescens* | *Vibrio cholerae* | *Haemophilus influenzae* | *Shewanella oneidensis* | *Pseudomonas aeruginosa* |

| | | | | | | | | |
|---|---|---|---|---|---|---|---|---|
| | *Salmonella typhimurium* | *Escherichia coli* | *Yersinia pestis KIM* | *Photorhabdus luminescens* | *Vibrio cholerae* | *Haemophilus influenzae* | *Shewanella oneidensis* | *Pseudomonas aeruginosa* |
| | *Salmonella enterica* | *Escherichia coli* | *Yersinia pestis KIM* | *Photorhabdus luminescens* | *Vibrio cholerae* | *Haemophilus influenzae* | *Shewanella oneidensis* | *Pseudomonas aeruginosa* |
| | *Escherichia coli* | *Yersinia pestis KIM* | *Salmonella enterica* | *Photorhabdus luminescens* | *Vibrio cholerae* | *Haemophilus influenzae* | *Shewanella oneidensis* | *Pseudomonas aeruginosa* |

# Table S3. Selenoprotein loss events identified in 25 bacterial selenoprotein families

| Selenoprotein | Phylum | Organism | Sister organisms | | | Distant organisms | | | |
|---|---|---|---|---|---|---|---|---|---|
| | | | 1 | 2 | 3 | 1 | 2 | 3 | 4 |
| FdhA | *Firmicutes/Clostridia/ Clostridium* | *Clostridium difficile* | *Clostridium perfringens (-)* | *Clostridium tetani (-)* | *Clostridium botulinum (-)* | *Thermoanaerobacter tengcongensis (-)* | *Moorella thermoacetica (U)* | *Desulfitobacterium hafniense (U)* | *Carboxydothermus hydrogenoformans (U)* |
| | *Firmicutes/Clostridia/ Thermoanaerobacteriales* | *Moorella thermoacetica* | *Thermoanaerobacter ethanolicus (-)* | *Thermoanaerobacter tengcongensis (-)* | *Ammonifex degensii (-)* | *Carboxydothermus hydrogenoformans (U)* | *Desulfitobacterium hafniense (U)* | *Clostridium difficile (U)* | *Desulfotomaculum reducens (U)* |
| SelD | *Actinobacteria* | *Symbiobacterium thermophilum* | *Rubrobacter xylanophilus (U)* | *Bifidobacterium longum (-)* | *Tropheryma whipplei (-)* | *Kineococcus radiotolerans (C)* | *Mycobacterium avium (C)* | *Brevibacterium linens (C)* | *Corynebacterium diphtheriae (-)* |
| | *Actinobacteria* | *Rubrobacter xylanophilus* | *Symbiobacterium thermophilum (U)* | *Bifidobacterium longum (-)* | *Tropheryma whipplei (-)* | *Kineococcus radiotolerans (C)* | *Mycobacterium avium (C)* | *Brevibacterium linens (C)* | *Corynebacterium diphtheriae (-)* |
| | *Firmicutes/Clostridia/ Clostridium* | *Clostridium difficile* | *Clostridium perfringens (U)* | *Clostridium tetani (-)* | *Clostridium botulinum (-)* | *Thermoanaerobacter tengcongensis (U)* | *Moorella thermoacetica (U)* | *Desulfitobacterium hafniense (C)* | *Carboxydothermus hydrogenoformans (U)* |
| | *Firmicutes/Clostridia/ Clostridium* | *Clostridium perfringens* | *Clostridium difficile (U)* | *Clostridium tetani (-)* | *Clostridium botulinum (-)* | *Thermoanaerobacter tengcongensis (U)* | *Moorella thermoacetica (U)* | *Desulfitobacterium hafniense (C)* | *Carboxydothermus hydrogenoformans (U)* |
| | *Firmicutes/Clostridia/ Thermoanaerobacteriales* | *Moorella thermoacetica* | *Thermoanaerobacter ethanolicus (-)* | *Thermoanaerobacter tengcongensis (U)* | *Ammonifex degensii (-)* | *Carboxydothermus hydrogenoformans (U)* | *Desulfitobacterium hafniense (C)* | *Clostridium difficile (U)* | *Desulfotomaculum reducens (U)* |
| | *Firmicutes/Clostridia/ Thermoanaerobacteriales* | *Thermoanaerobacter tengcongensis* | *Moorella thermoacetica (U)* | *Thermoanaerobacter ethanolicus (-)* | *Ammonifex degensii (-)* | *Carboxydothermus hydrogenoformans (U)* | *Desulfitobacterium hafniense (C)* | *Clostridium difficile (U)* | *Desulfotomaculum reducens (U)* |
| FrhD | *Firmicutes/Clostridia/ Thermoanaerobacteriales* | *Moorella thermoacetica* | *Thermoanaerobacter ethanolicus (-)* | *Thermoanaerobacter tengcongensis (-)* | *Ammonifex degensii (-)* | *Clostridium difficile (-)* | *Desulfotomaculum reducens (U)* | *Desulfitobacterium hafniense (-)* | *Carboxydothermus hydrogenoformans (U)* |
| | *Firmicutes/Clostridia/Peptococcaceae* | *Carboxydothermus hydrogenoformans* | *Desulfotomaculum reducens (U)* | *Desulfitobacterium hafniense (-)* | | *Moorella thermoacetica (U)* | *Syntrophomonas wolfei (U)* | *Clostridium difficile (-)* | *Thermoanaerobacter tengcongensis (-)* |
| | *Firmicutes/Clostridia/Peptococcaceae* | *Desulfotomaculum reducens (2)* | *Carboxydothermus hydrogenoformans (U)* | *Desulfitobacterium hafniense (-)* | | *Moorella thermoacetica (U)* | *Syntrophomonas wolfei (U)* | *Clostridium difficile (-)* | *Thermoanaerobacter tengcongensis (-)* |
| | *Firmicutes/Clostridia/Syntrophomonadaceae* | *Syntrophomonas wolfei* | *Caldicellulosiruptor saccharolyticus (-)* | *Anaerobranca gottschalkii (-)* | | *Moorella thermoacetica (U)* | *Carboxydothermus hydrogenoformans (U)* | *Thermoanaerobacter tengcongensis (-)* | *Desulfotomaculum reducens (U)* |
| HdrA | *Firmicutes/Clostridia/ Thermoanaerobacteriales* | *Moorella thermoacetica* | *Thermoanaerobacter ethanolicus (-)* | *Thermoanaerobacter tengcongensis (-)* | *Ammonifex degensii (-)* | *Clostridium difficile (-)* | *Syntrophomonas wolfei (U)* | *Desulfotomaculum reducens (U)* | *Carboxydothermus hydrogenoformans (U)* |
| | *Firmicutes/Clostridia/Peptococcaceae* | *Carboxydothermus hydrogenoformans* | *Desulfotomaculum reducens (U)* | *Desulfitobacterium hafniense (-)* | | *Moorella thermoacetica (U)* | *Syntrophomonas wolfei (U)* | *Clostridium difficile (-)* | *Thermoanaerobacter tengcongensis (-)* |
| | *Firmicutes/Clostridia/Peptococcaceae* | *Desulfotomaculum reducens* | *Carboxydothermus hydrogenoformans (U)* | *Desulfitobacterium hafniense (-)* | | *Moorella thermoacetica (U)* | *Syntrophomonas wolfei (U)* | *Clostridium difficile (-)* | *Thermoanaerobacter tengcongensis (-)* |
| | *Firmicutes/Clostridia/Syntrophomonadaceae* | *Syntrophomonas wolfei* | *Caldicellulosiruptor saccharolyticus (-)* | *Anaerobranca gottschalkii (-)* | | *Moorella thermoacetica (U)* | *Carboxydothermus hydrogenoformans (U)* | *Thermoanaerobacter tengcongensis (-)* | *Desulfotomaculum reducens (-)* |
| HesB-like | *Actinobacteria* | *Symbiobacterium thermophilum* | *Rubrobacter xylanophilus (-)* | *Bifidobacterium longum (-)* | *Tropheryma whipplei (-)* | *Kineococcus radiotolerans (C)* | *Mycobacterium avium (C)* | *Brevibacterium linens (C)* | *Corynebacterium diphtheriae (C)* |
| | *Deltaproteobacteria/ Geobacteraceae* | *Geobacter sulfurreducens* | *Geobacter metallireducens (-)* | *Geobacter uraniumreducens (-)* | *Pelobacter carbinolicus (-)* | *Syntrophobacter fumaroxidans (U)* | *Desulfovibrio vulgaris (U)* | *Syntrophus aciditrophicus (U)* | *Desulfuromonas acetoxidans (U)* |
| | *Firmicutes/Clostridia/Peptococcaceae* | *Desulfitobacterium hafniense* | *Carboxydothermus hydrogenoformans (-)* | *Desulfotomaculum reducens (-)* | | *Clostridium perfringens (U)* | *Alkaliphilus metalliredigenes (U)* | *Thermoanaerobacter tengcongensis (-)* | *Moorella thermoacetica (-)* |
| GrdA | *Firmicutes/Clostridia/Peptococcaceae* | *Carboxydothermus hydrogenoformans* | *Desulfotomaculum reducens (-)* | *Desulfitobacterium hafniense (-)* | | *Thermoanaerobacter tengcongensis (U)* | *Clostridium difficile (U)* | *Moorella thermoacetica (-)* | *Syntrophomonas wolfei (-)* |

| | | | | | | | | | |
|---|---|---|---|---|---|---|---|---|---|
| | *Firmicutes/Clostridia/Thermoanaerobacteriales* | *Thermoanaerobacter tengcongensis* | *Moorella thermoacetica (-)* | *Thermoanaerobacter ethanolicus (-)* | *Ammonifex degensii (-)* | *Carboxydothermus hydrogenoformans (U)* | *Clostridium difficile (U)* | *Desulfitobacterium hafniense (-)* | *Desulfotomaculum reducens (-)* |
| | *Firmicutes/Clostridia/ Clostridium* | *Clostridium difficile* | *Clostridium perfringens (-)* | *Clostridium tetani (-)* | *Clostridium botulinum (-)* | *Moorella thermoacetica (-)* | *Carboxydothermus hydrogenoformans (U)* | *Thermoanaerobacter tengcongensis (U)* | *Desulfitobacterium hafniense (-)* |
| | *Firmicutes/Clostridia* | *Alkaliphilus metalliredigenes* | *Clostridium difficile (U)* | *Clostridium perfringens (-)* | *Clostridium tetani (-)* | *Carboxydothermus hydrogenoformans (U)* | *Thermoanaerobacter tengcongensis (U)* | *Desulfotomaculum reducens (-)* | *Moorella thermoacetica (-)* |
| **GrdB** | *Deltaproteobacteria/ Syntrophobacterales* | *Syntrophus aciditrophicus* | *Syntrophus buswellii (-)* | *Syntrophobacter fumaroxidans (-)* | | *Desulfuromonas acetoxidans (U)* | *Desulfotalea psychrophila (-)* | *Desulfovibrio vulgaris (-)* | *Geobacter sulfurreducens (-)* |
| | *Deltaproteobacteria/ Desulfuromonadales* | *Desulfuromonas acetoxidans* | *Geobacter metallireducens (-)* | *Geobacter sulfurreducens (-)* | *Pelobacter carbinolicus (-)* | *Syntrophus aciditrophicus (U)* | *Desulfotalea psychrophila (-)* | *Anaeromyxobacter dehalogenans (-)* | *Desulfovibrio vulgaris (-)* |
| | *Firmicutes/Clostridia/Peptococcaceae* | *Carboxydothermus hydrogenoformans* | *Desulfotomaculum reducens (-)* | *Desulfitobacterium hafniense (-)* | | *Thermoanaerobacter tengcongensis (U)* | *Clostridium difficile (U)* | *Moorella thermoacetica (-)* | *Syntrophomonas wolfei (-)* |
| | *Firmicutes/Clostridia/Thermoanaerobacteriales* | *Thermoanaerobacter tengcongensis* | *Moorella thermoacetica (-)* | *Thermoanaerobacter ethanolicus (-)* | *Ammonifex degensii (-)* | *Carboxydothermus hydrogenoformans (U)* | *Clostridium difficile (U)* | *Desulfitobacterium hafniense (-)* | *Desulfotomaculum reducens (-)* |
| | *Firmicutes/Clostridia/ Clostridium* | *Clostridium difficile* | *Clostridium perfringens (-)* | *Clostridium tetani (-)* | *Clostridium botulinum (-)* | *Carboxydothermus hydrogenoformans (U)* | *Thermoanaerobacter tengcongensis (U)* | *Moorella thermoacetica (-)* | *Desulfitobacterium hafniense (-)* |
| | *Firmicutes/Clostridia* | *Alkaliphilus metalliredigenes* | *Clostridium difficile (U)* | *Clostridium perfringens (-)* | *Clostridium tetani (-)* | *Carboxydothermus hydrogenoformans (U)* | *Thermoanaerobacter tengcongensis (U)* | *Desulfotomaculum reducens (-)* | *Moorella thermoacetica (-)* |
| **SelW-like** | *Actinobacteria* | *Symbiobacterium thermophilum* | *Rubrobacter xylanophilus (-)* | *Bifidobacterium longum (-)* | *Tropheryma whipplei (-)* | *Kineococcus radiotolerans (C)* | *Streptomyces avermitilis (C)* | *Mycobacterium avium (-)* | *Corynebacterium diphtheriae (-)* |
| | *Deltaproteobacteria/ Geobacteraceae* | *Geobacter sulfurreducens* | *Geobacter metallireducens (-)* | *Geobacter uraniumreducens (-)* | *Pelobacter carbinolicus (-)* | *Desulfotalea psychrophila (U)* | *Desulfovibrio vulgaris (-)* | *Syntrophus aciditrophicus (-)* | *Desulfuromonas acetoxidans (C)* |
| | *Deltaproteobacteria/Desulfobacterales* | *Desulfotalea psychrophila* | *Desulfobacterium autotrophicum (-)* | *Nitrospina gracilis (-)* | | *Geobacter sulfurreducens (U)* | *Desulfuromonas acetoxidans (C)* | *Desulfovibrio vulgaris (-)* | *Anaeromyxobacter dehalogenans (-)* |
| **DsbA-like** | *Firmicutes/Clostridia/Peptococcaceae* | *Desulfotomaculum reducens* | *Carboxydothermus hydrogenoformans (-)* | *Desulfitobacterium hafniense (-)* | | *Clostridium difficile (C)* | *Thermoanaerobacter tengcongensis (C)* | *Moorella thermoacetica (-)* | *Syntrophomonas wolfei (-)* |
| **ArsC** | *Deltaproteobacteria/ Desulfuromonadales* | *Desulfuromonas acetoxidans* | *Geobacter metallireducens (-)* | *Geobacter sulfurreducens (-)* | *Pelobacter carbinolicus (-)* | *Desulfotalea psychrophila (U)* | *Syntrophus aciditrophicus (-)* | *Anaeromyxobacter dehalogenans (-)* | *Desulfovibrio vulgaris (-)* |
| | *Deltaproteobacteria/Desulfobacterales* | *Desulfotalea psychrophila* | *Desulfobacterium autotrophicum (-)* | *Nitrospina gracilis (-)* | | *Desulfuromonas acetoxidans (U)* | *Anaeromyxobacter dehalogenans (-)* | *Geobacter sulfurreducens (-)* | *Desulfovibrio vulgaris (-)* |
| **AhpF** | *Firmicutes/Clostridia/Peptococcaceae* | *Carboxydothermus hydrogenoformans* | *Desulfotomaculum reducens (-)* | *Desulfitobacterium hafniense (C)* | | *Clostridium difficile (C)* | *Moorella thermoacetica (-)* | *Syntrophomonas wolfei (-)* | *Thermoanaerobacter tengcongensis (-)* |
| **NADH oxidase** | *Deltaproteobacteria/ Geobacteraceae* | *Geobacter metallireducens* | *Geobacter sulfurreducens (C)* | *Geobacter uraniumreducens (-)* | *Pelobacter carbinolicus (-)* | *Desulfotalea psychrophila (C)* | *Desulfovibrio vulgaris (C)* | *Syntrophus aciditrophicus (C)* | *Desulfuromonas acetoxidans (-)* |

**Table S4. Relationship between selenium utilization traits and oxygen requirement**

| | Sec trait (only) | Sec and Selenouridine traits | Selenouridine trait | No selenium utilization trait s | Total |
|---|---|---|---|---|---|
| **Anaerobic** | 13 | 11 | 5 | 27 | 56 |
| **Facultative** | 12 | 14 | 17 | 73 | 116 |
| **Microaerophilic** | 3 | 6 | 2 | 21 | 32 |
| **Aerobic** | 5 | 11 | 22 | 107 | 145 |
| **Total** | 33 | 42 | 46 | 228 | 349 |

**Table S5. Relationship between selenium utilization traits and optimal growth temperature**

| | Sec trait (only) | Sec and Selenouridine traits | Selenouridine trait | No selenium utilization trait s | Total |
|---|---|---|---|---|---|
| **< 20 °C (Psychrophilic)** | 0 | 2 | 3 | 6 | 11 |
| **20~30 °C** | 13 | 17 | 40 | 117 | 187 |
| **30~40 °C** | 15 | 21 | 3 | 93 | 132 |
| **> 40 °C (Thermophilic)** | 5 | 2 | 0 | 12 | 19 |
| **Total** | 33 | 42 | 46 | 228 | 349 |

**Table S6. Relationship between Sec-/Cys-containing Prx and oxygen requirement**

| | Prx (Sec) | Prx (Cys in Sec$^{+}$)* | Prx (Cys in Sec$^{-}$)* | Prx lacking | Total |
|---|---|---|---|---|---|
| **Anaerobic** | 7 | 10 | 33 | 6 | 56 |
| **Facultative** | 2 | 15 | 90 | 9 | 116 |
| **Microaerophilic** | 0 | 14 | 6 | 12 | 32 |
| **Aerobic** | 1 | 17 | 122 | 5 | 145 |
| **Total** | **10** | **56** | **251** | **32** | **349** |

Sec$^{+}$: Organisms containing the Sec-decoding trait

Sec$^{-}$: Organisms lacking the Sec-decoding trait

**Table S7. Relationship between Sec-/Cys-containing HesB-like and oxygen requirement**

| | HesB-like (Sec) | HesB-like (Cys in Sec$^{+}$) | HesB-like (Cys in Sec$^{-}$) | HesB-like lacking | Total |
|---|---|---|---|---|---|
| **Anaerobic** | 7 | 5 | 3 | 41 | 56 |
| **Facultative** | 2 | 4 | 14 | 96 | 116 |
| **Microaerophilic** | 2 | 5 | 20 | 5 | 32 |
| **Aerobic** | 0 | 6 | 26 | 113 | 145 |
| **Total** | **11** | **20** | **63** | **255** | **349** |
