# Supplementary material for: Dynamic evolution of selenocysteine utilization in bacteria: a balance between selenoprotein loss and evolution of selenocysteine from redox active cysteine residues
Source: Genome Biol. 2006 Oct 20;7(10):R94. doi: 10.1186/gb-2006-7-10-r94 (PMC1794560; doi:10.1186/gb-2006-7-10-r94)
Supplement: Additional data file 2 — Figure S1 shows phylograms of SelA, SelB, SelD, and YbbB sequences. Figures S2 and S3 show the distribution of selenoproteins and their Cys-containing homologs in different organisms. [file gb-2006-7-10-r94-S2.pdf]

# Figure Legends

**Fig. S1. Phylograms of SelA, SelB, SelD and YbbB sequences.** A total of 75 SelA, 75 SelB, 127 SelD and 88 YbbB sequences were identified in 349 completely and incompletely sequenced bacterial genomes. Organisms and phyla are separated using different shading patterns. In the SelD phylogram, U represents Sec-containing sequences and C represents Cys-containing sequences. A. SelA; B. SelB; C. SelD; D. YbbB.

**Fig. S2. Distribution of selenoproteins and their Cys-containing homologs in organisms with and without the Sec trait.** For each selenoprotein family, the occurrence of selenoproteins and their Cys homologs was determined in organisms with the Sec trait ($Sec^{+}$) and of the Cys homologs in organisms lacking the Sec trait ($Sec^{-}$). The data for each family are shown in three columns: (i) selenoproteins (Sec in $Sec^{+}$, shown in red), (ii) Cys-containing homologs and the Sec trait (Cys in $Sec^{+}$, shown in green), and (iii) Cys-containing homologs and no Sec trait (Cys in $Sec^{-}$, shown in blue).

**Fig. S3. Distribution of selenoproteins and their Cys-containing homologs in Sec-containing organisms.** Two selenoprotein families (ArsC-like and DsrE-like) were excluded because the total number of both Sec- and Cys-containing sequences in Sec-containing organisms was low (less than 5). Remaining selenoprotein families were ordered based on the percentage of Sec and Cys forms of these proteins present in organisms with the Sec trait. Red, Sec in $Sec^{+}$; Green, Cys in $Sec^{+}$.

**Fig. S1**

**A. SelA**

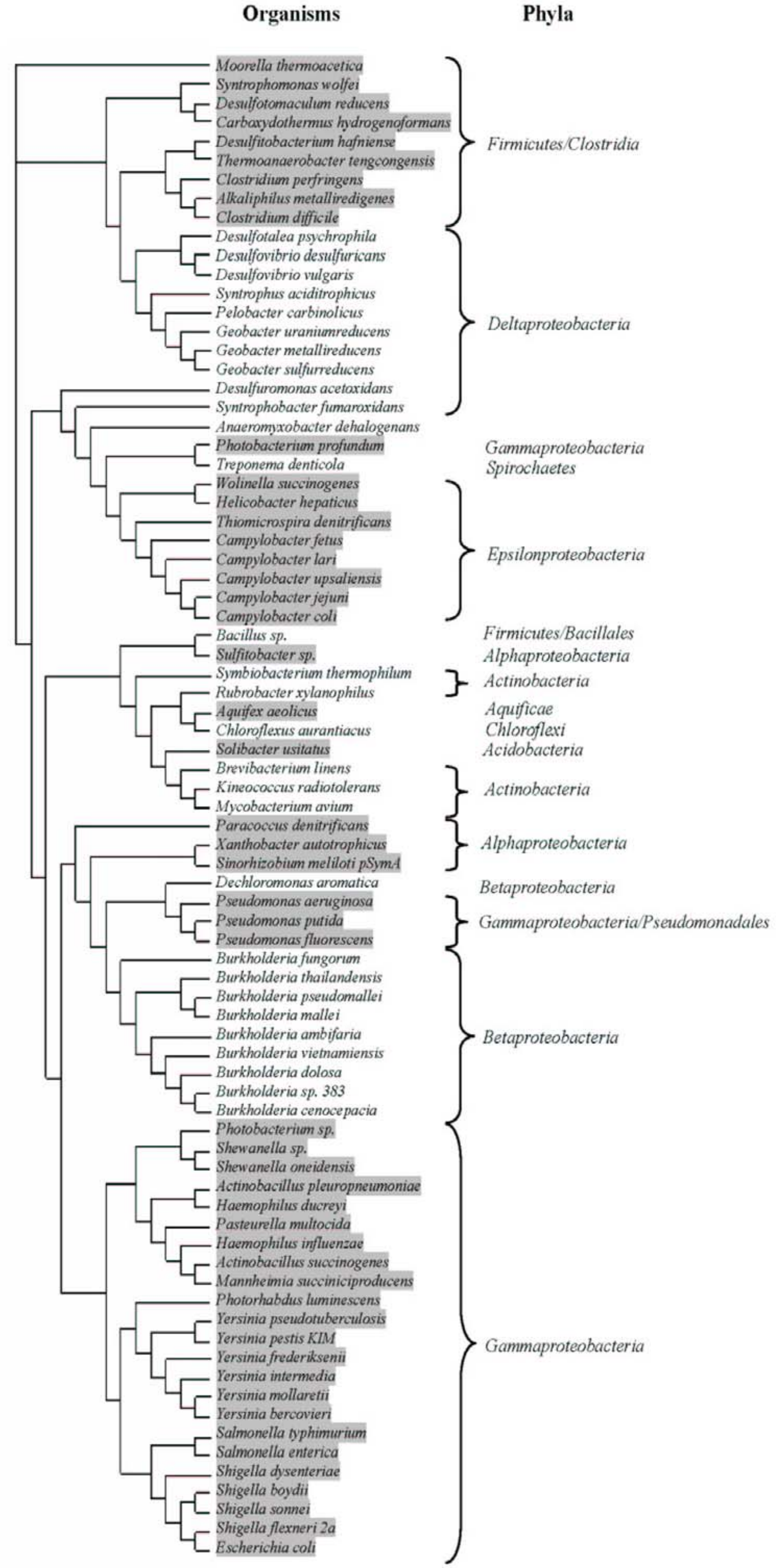
Organisms
Phyla
Moorella thermoacetica
Syntrophomonas wolfei
Desulfotomaculum reducens
Carboxydothermus hydrogenoformans
Desulfitobacterium hafniense
Thermoanaerobacter tengcongensis
Clostridium perfringens
Alkaliphilus metalliredigenes
Clostridium difficile
Firmicutes/Clostridia
Desulfotalea psychrophila
Desulfovibrio desulfuricans
Desulfovibrio vulgaris
Syntrophus aciditrophicus
Pelobacter carbinolicus
Geobacter uraniumreducens
Geobacter metallireducens
Geobacter sulfurreducens
Desulfuromonas acetoxidans
Syntrophobacter fumaroxidans
Deltaproteobacteria
Anaeromyxobacter dehalogenans
Photobacterium profundum
Treponema denticola
Gammaproteobacteria
Spirochaetes
Wolinella succinogenes
Helicobacter hepaticus
Thiomicrospira denitrificans
Campylobacter fetus
Campylobacter lari
Campylobacter upsaliensis
Campylobacter jejuni
Campylobacter coli
Epsilonproteobacteria
Bacillus sp.
Sulfitobacter sp.
Symbiobacterium thermophilum
Rubrobacter xylanophilus
Aquifex aeolicus
Chloroflexus aurantiacus
Solibacter usitatus
Firmicutes/Bacillales
Alphaproteobacteria
Actinobacteria
Aquificae
Chloroflexi
Acidobacteria
Brevibacterium linens
Kineococcus radiotolerans
Mycobacterium avium
Actinobacteria
Paracoccus denitrificans
Xanthobacter autotrophicus
Sinorhizobium meliloti pSymA
Alphaproteobacteria
Dechloromonas aromatica
Betaproteobacteria
Pseudomonas aeruginosa
Pseudomonas putida
Pseudomonas fluorescens
Gammaproteobacteria/Pseudomonadales
Burkholderia fungorum
Burkholderia thailandensis
Burkholderia pseudomallei
Burkholderia mallei
Burkholderia ambifaria
Burkholderia vietnamiensis
Burkholderia dolosa
Burkholderia sp. 383
Burkholderia cenocepacia
Betaproteobacteria
Photobacterium sp.
Shewanella sp.
Shewanella oneidensis
Actinobacillus pleuropneumoniae
Haemophilus ducreyi
Pasteurella multocida
Haemophilus influenzae
Actinobacillus succinogenes
Mannheimia succiniciproducens
Photorhabdus luminescens
Yersinia pseudotuberculosis
Yersinia pestis KIM
Yersinia frederiksenii
Yersinia intermedia
Yersinia mollaretii
Yersinia bercovieri
Salmonella typhimurium
Salmonella enterica
Shigella dysenteriae
Shigella boydii
Shigella sonnei
Shigella flexneri 2a
Escherichia coli
Gammaproteobacteria

**B. SelB**

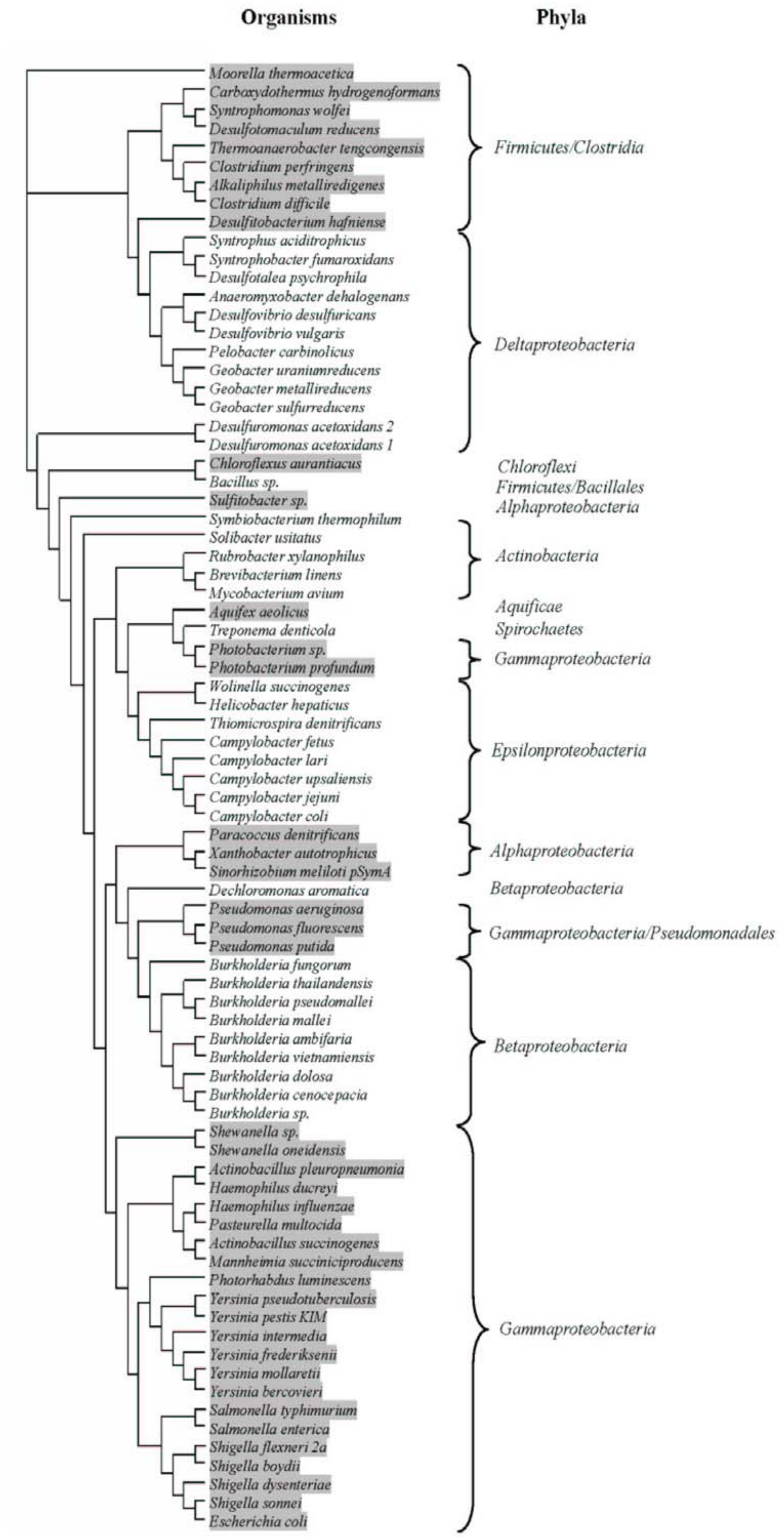
Organisms
Phyla
Moorella thermoacetica
Carboxydothermus hydrogenoformans
Syntrophomonas wolfei
Desulfotomaculum reducens
Thermoanaerobacter tengcongensis
Clostridium perfringens
Alkaliphilus metalliredigenes
Clostridium difficile
Desulfitobacterium hafniense
Firmicutes/Clostridia
Syntrophus aciditrophicus
Syntrophobacter fumaroxidans
Desulfotalea psychrophila
Anaeromyxobacter dehalogenans
Desulfovibrio desulfuricans
Desulfovibrio vulgaris
Pelobacter carbinolicus
Geobacter uraniumreducens
Geobacter metallireducens
Geobacter sulfurreducens
Desulfuromonas acetoxidans 2
Desulfuromonas acetoxidans 1
Deltaproteobacteria
Chloroflexus aurantiacus
Chloroflexi
Bacillus sp.
Firmicutes/Bacillales
Sulfitobacter sp.
Alphaproteobacteria
Symbiobacterium thermophilum
Solibacter usitatus
Rubrobacter xylanophilus
Brevibacterium linens
Mycobacterium avium
Actinobacteria
Aquifex aeolicus
Aquificae
Treponema denticola
Spirochaetes
Photobacterium sp.
Photobacterium profundum
Gammaproteobacteria
Wolinella succinogenes
Helicobacter hepaticus
Thiomicrospira denitrificans
Campylobacter fetus
Campylobacter lari
Campylobacter upsaliensis
Campylobacter jejuni
Campylobacter coli
Epsilonproteobacteria
Paracoccus denitrificans
Xanthobacter autotrophicus
Sinorhizobium meliloti pSymA
Alphaproteobacteria
Dechloromonas aromatica
Betaproteobacteria
Pseudomonas aeruginosa
Pseudomonas fluorescens
Pseudomonas putida
Gammaproteobacteria/Pseudomonadales
Burkholderia fungorum
Burkholderia thailandensis
Burkholderia pseudomallei
Burkholderia mallei
Burkholderia ambifaria
Burkholderia vietnamiensis
Burkholderia dolosa
Burkholderia cenocepacia
Burkholderia sp.
Betaproteobacteria
Shewanella sp.
Shewanella oneidensis
Actinobacillus pleuropneumonia
Haemophilus ducreyi
Haemophilus influenzae
Pasteurella multocida
Actinobacillus succinogenes
Mannheimia succiniciproducens
Photorhabdus luminescens
Yersinia pseudotuberculosis
Yersinia pestis KIM
Yersinia intermedia
Yersinia frederiksenii
Yersinia mollaretii
Yersinia bercovieri
Salmonella typhimurium
Salmonella enterica
Shigella flexneri 2a
Shigella boydii
Shigella dysenteriae
Shigella sonnei
Escherichia coli
Gammaproteobacteria

## C. SelD

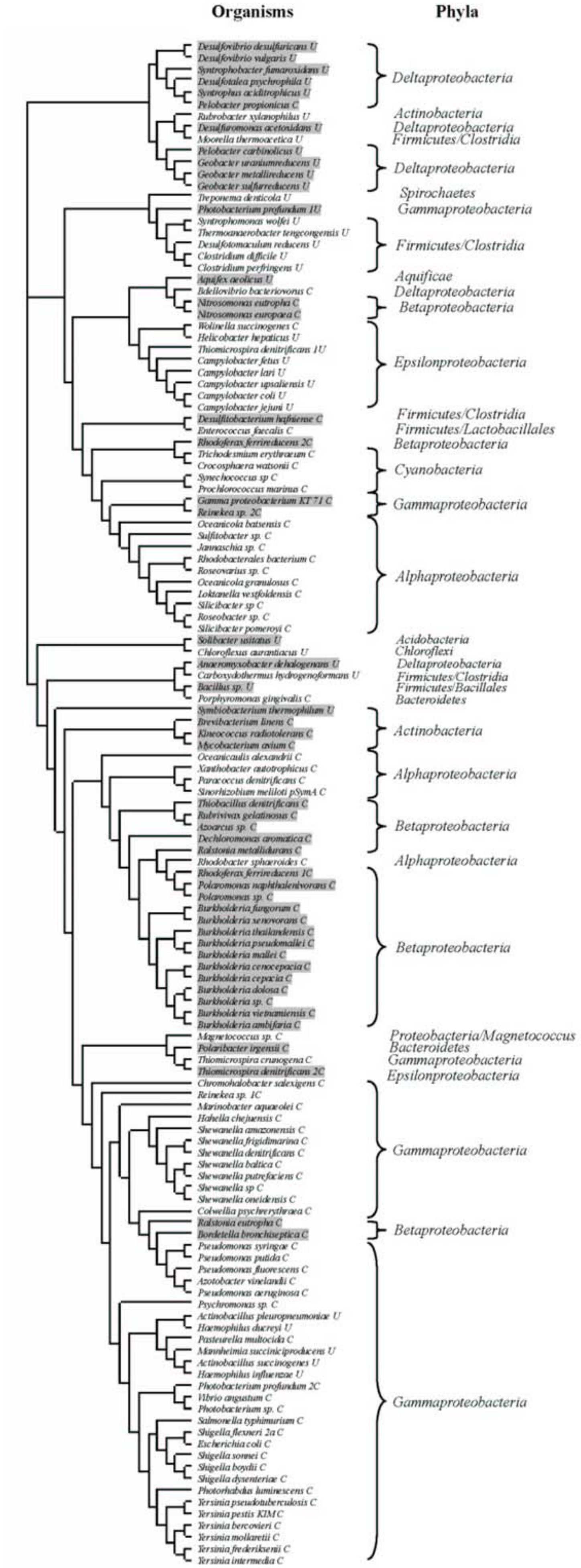

Organisms
Phyla
Desulfovibrio desulfuricans U
Desulfovibrio vulgaris U
Syntrophobacter fumaroxidans U
Desulfotalea psychrophila U
Syntrophus aciditrophicus U
Pelobacter propionicus C
Deltaproteobacteria
Rubrobacter xylanophilus U
Actinobacteria
Desulfuromonas acetoxidans U
Deltaproteobacteria
Moorella thermoacetica U
Firmicutes/Clostridia
Pelobacter carbinolicus U
Geobacter uraniumreducens U
Geobacter metallireducens U
Geobacter sulfurreducens U
Deltaproteobacteria
Treponema denticola U
Spirochaetes
Photobacterium profundum 1U
Gammaproteobacteria
Syntrophomonas wolfei U
Thermoanaerobacter tengcongensis U
Desulfotomaculum reducens U
Clostridium difficile U
Clostridium perfringens U
Firmicutes/Clostridia
Aquifex aeolicus U
Aquificae
Bdellovibrio bacteriovorus C
Deltaproteobacteria
Nitrosomonas eutropha C
Nitrosomonas europaea C
Betaproteobacteria
Wolinella succinogenes C
Helicobacter hepaticus U
Thiomicrospira denitrificans 1U
Campylobacter fetus U
Campylobacter lari U
Campylobacter upsaliensis U
Campylobacter coli U
Campylobacter jejuni U
Epsilonproteobacteria
Desulfitobacterium hafniense C
Firmicutes/Clostridia
Enterococcus faecalis C
Firmicutes/Lactobacillales
Rhodoferax ferrireducens 2C
Betaproteobacteria
Trichodesmium erythraeum C
Crocosphaera watsonii C
Synechococcus sp C
Prochlorococcus marinus C
Cyanobacteria
Gamma proteobacterium KT 71 C
Reinekea sp. 2C
Gammaproteobacteria
Oceanicola batsensis C
Sulfitobacter sp. C
Jannaschia sp. C
Rhodobacterales bacterium C
Roseovarius sp. C
Oceanicola granulosus C
Loktanella vestfoldensis C
Silicibacter sp C
Roseobacter sp. C
Silicibacter pomeroyi C
Alphaproteobacteria
Solibacter usitatus U
Acidobacteria
Chloroflexus aurantiacus U
Chloroflexi
Anaeromyxobacter dehalogenans U
Deltaproteobacteria
Carboxydothermus hydrogenoformans U
Firmicutes/Clostridia
Bacillus sp. U
Firmicutes/Bacillales
Porphyromonas gingivalis C
Bacteroidetes
Symbiobacterium thermophilum U
Brevibacterium linens C
Kineococcus radiotolerans C
Mycobacterium avium C
Actinobacteria
Oceanicaulis alexandrii C
Xanthobacter autotrophicus C
Paracoccus denitrificans C
Sinorhizobium meliloti pSymA C
Alphaproteobacteria
Thiobacillus denitrificans C
Rubrivivax gelatinosus C
Azoarcus sp. C
Dechloromonas aromatica C
Ralstonia metallidurans C
Betaproteobacteria
Rhodobacter sphaeroides C
Alphaproteobacteria
Rhodoferax ferrireducens 1C
Polaromonas naphthalenivorans C
Polaromonas sp. C
Burkholderia fungorum C
Burkholderia xenovorans C
Burkholderia thailandensis C
Burkholderia pseudomallei C
Burkholderia mallei C
Burkholderia cenocepacia C
Burkholderia cepacia C
Burkholderia dolosa C
Burkholderia sp. C
Burkholderia vietnamiensis C
Burkholderia ambifaria C
Betaproteobacteria
Magnetococcus sp. C
Proteobacteria/Magnetococcus
Polaribacter irgensii C
Bacteroidetes
Thiomicrospira crunogena C
Gammaproteobacteria
Thiomicrospira denitrificans 2C
Epsilonproteobacteria
Chromohalobacter salexigens C
Reinekea sp. 1C
Marinobacter aquaeolei C
Hahella chejuensis C
Shewanella amazonensis C
Shewanella frigidimarina C
Shewanella denitrificans C
Shewanella baltica C
Shewanella putrefaciens C
Shewanella sp C
Shewanella oneidensis C
Colwellia psychrerythraea C
Gammaproteobacteria
Ralstonia eutropha C
Bordetella bronchiseptica C
Betaproteobacteria
Pseudomonas syringae C
Pseudomonas putida C
Pseudomonas fluorescens C
Azotobacter vinelandii C
Pseudomonas aeruginosa C
Psychromonas sp. C
Actinobacillus pleuropneumoniae U
Haemophilus ducreyi U
Pasteurella multocida C
Mannheimia succiniciproducens U
Actinobacillus succinogenes U
Haemophilus influenzae U
Photobacterium profundum 2C
Vibrio angustum C
Photobacterium sp. C
Salmonella typhimurium C
Shigella flexneri 2a C
Escherichia coli C
Shigella sonnei C
Shigella boydii C
Shigella dysenteriae C
Photorhabdus luminescens C
Yersinia pseudotuberculosis C
Yersinia pestis KIM C
Yersinia bercovieri C
Yersinia mollaretii C
Yersinia frederiksenii C
Yersinia intermedia C
Gammaproteobacteria

**D. YbbB**

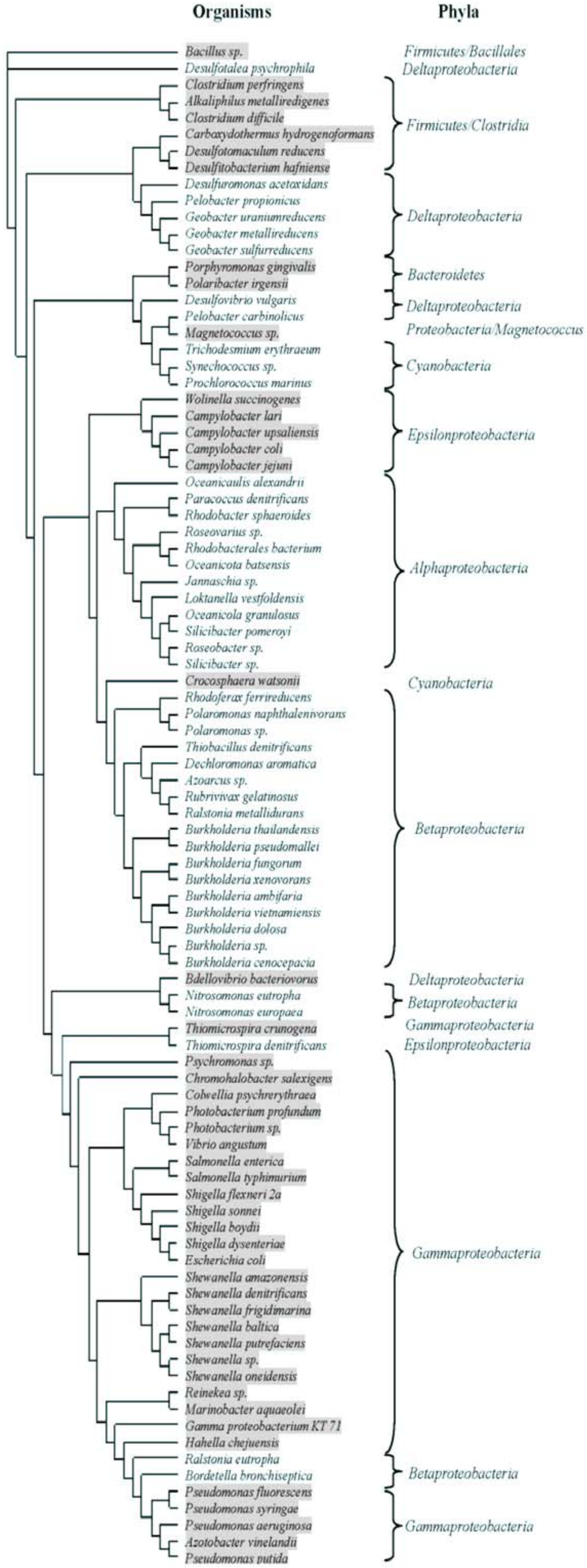

Organisms
Phyla
Bacillus sp.
Desulfotalea psychrophila
Clostridium perfringens
Alkaliphilus metalliredigenes
Clostridium difficile
Carboxydothermus hydrogenoformans
Desulfotomaculum reducens
Desulfitobacterium hafniense
Desulfuromonas acetoxidans
Pelobacter propionicus
Geobacter uraniumreducens
Geobacter metallireducens
Geobacter sulfurreducens
Porphyromonas gingivalis
Polaribacter irgensii
Desulfovibrio vulgaris
Pelobacter carbinolicus
Magnetococcus sp.
Trichodesmium erythraeum
Synechococcus sp.
Prochlorococcus marinus
Wolinella succinogenes
Campylobacter lari
Campylobacter upsaliensis
Campylobacter coli
Campylobacter jejuni
Oceanicaulis alexandrii
Paracoccus denitrificans
Rhodobacter sphaeroides
Roseovarius sp.
Rhodobacterales bacterium
Oceanicota batsensis
Jannaschia sp.
Loktanella vestfoldensis
Oceanicola granulosus
Silicibacter pomeroyi
Roseobacter sp.
Silicibacter sp.
Crocosphaera watsonii
Rhodoferax ferrireducens
Polaromonas naphthalenivorans
Polaromonas sp.
Thiobacillus denitrificans
Dechloromonas aromatica
Azoarcus sp.
Rubrivivax gelatinosus
Ralstonia metallidurans
Burkholderia thailandensis
Burkholderia pseudomallei
Burkholderia fungorum
Burkholderia xenovorans
Burkholderia ambifaria
Burkholderia vietnamiensis
Burkholderia dolosa
Burkholderia sp.
Burkholderia cenocepacia
Bdellovibrio bacteriovorus
Nitrosomonas eutropha
Nitrosomonas europaea
Thiomicrospira crunogena
Thiomicrospira denitrificans
Psychromonas sp.
Chromohalobacter salexigens
Colwellia psychrerythraea
Photobacterium profundum
Photobacterium sp.
Vibrio angustum
Salmonella enterica
Salmonella typhimurium
Shigella flexneri 2a
Shigella sonnei
Shigella boydii
Shigella dysenteriae
Escherichia coli
Shewanella amazonensis
Shewanella denitrificans
Shewanella frigidimarina
Shewanella baltica
Shewanella putrefaciens
Shewanella sp.
Shewanella oneidensis
Reinekea sp.
Marinobacter aquaeolei
Gamma proteobacterium KT 71
Hahella chejuensis
Ralstonia eutropha
Bordetella bronchiseptica
Pseudomonas fluorescens
Pseudomonas syringae
Pseudomonas aeruginosa
Azotobacter vinelandii
Pseudomonas putida
Firmicutes/Bacillales
Deltaproteobacteria
Firmicutes/Clostridia
Deltaproteobacteria
Bacteroidetes
Deltaproteobacteria
Proteobacteria/Magnetococcus
Cyanobacteria
Epsilonproteobacteria
Alphaproteobacteria
Cyanobacteria
Betaproteobacteria
Deltaproteobacteria
Betaproteobacteria
Gammaproteobacteria
Epsilonproteobacteria
Gammaproteobacteria
Betaproteobacteria
Gammaproteobacteria

**Fig. S2**

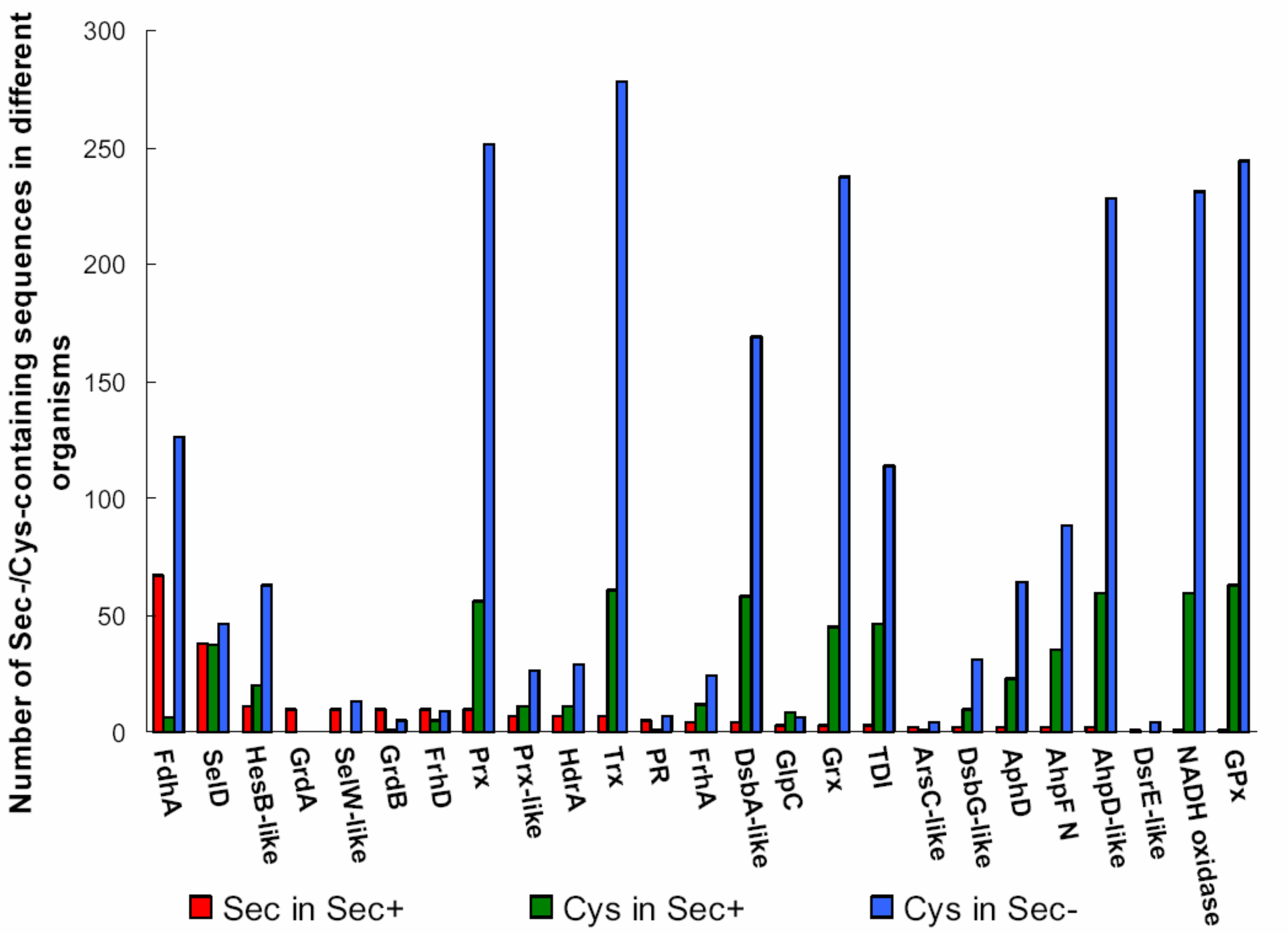
Number of Sec-/Cys-containing sequences in different organisms
300
250
200
150
100
50
0
FdhA
SelD
HesB-like
GrdA
SelW-like
GrdB
FrhD
Prx
Prx-like
HdrA
Trx
PR
FrhA
DsbA-like
GlpC
Grx
TDI
ArsC-like
DsbG-like
AphD
AhpF N
AhpD-like
DsrE-like
NADH oxidase
GPx
Sec in Sec+
Cys in Sec+
Cys in Sec-

**Fig. S3**

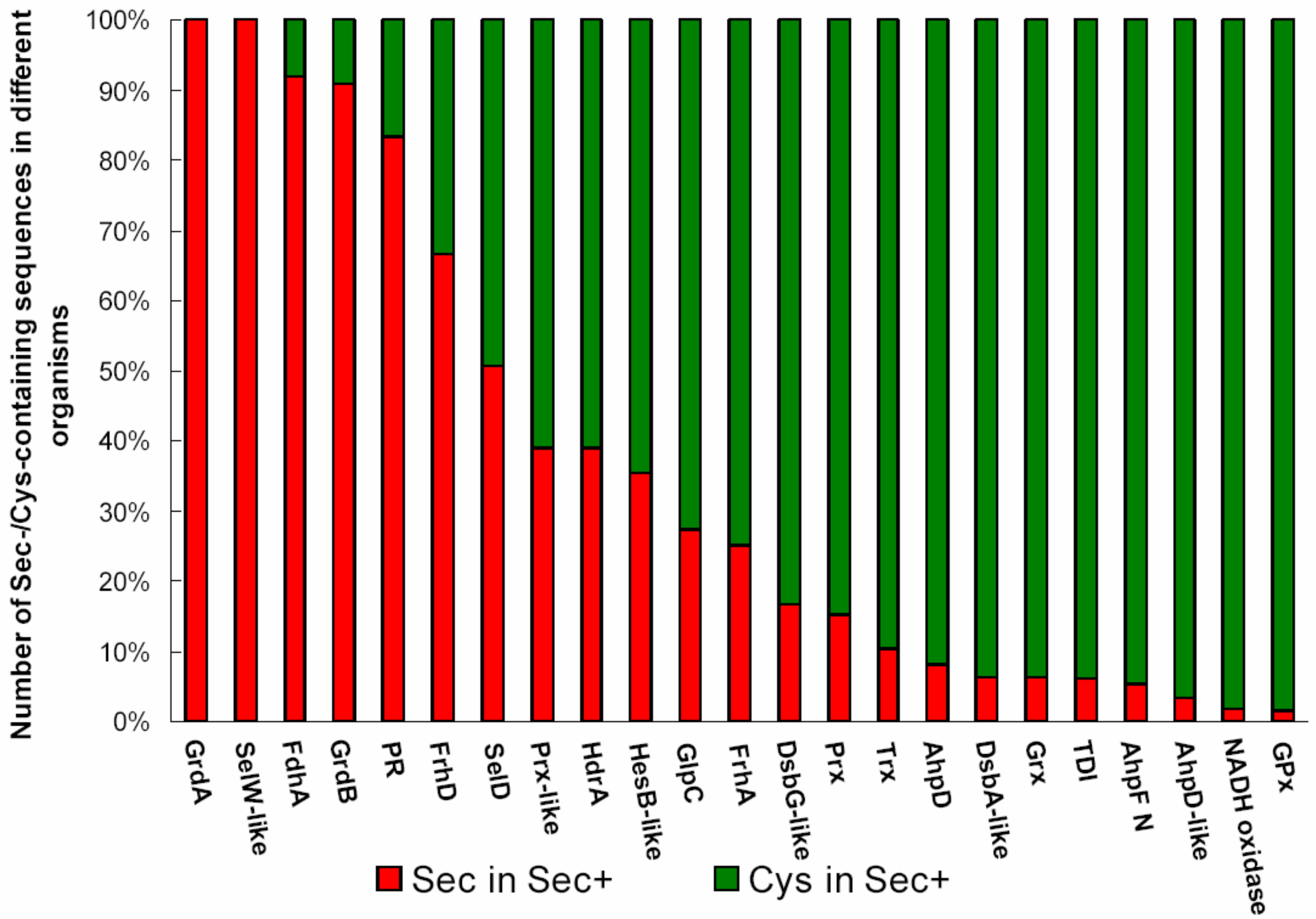

Number of Sec-/Cys-containing sequences in different organisms
100%
90%
80%
70%
60%
50%
40%
30%
20%
10%
0%
GrdA
SelW-like
FdhA
GrdB
PR
FrhD
SelD
Prx-like
HdrA
HesB-like
GlpC
FrhA
DsbG-like
Prx
Trx
AhpD
DsbA-like
Grx
TDI
AhpF N
AhpD-like
NADH oxidase
GPx
Sec in Sec+
Cys in Sec+
